# Optimisation of 16S rRNA gut microbiota profiling of extremely low birth weight infants

**DOI:** 10.1186/s12864-017-4229-x

**Published:** 2017-11-02

**Authors:** Cristina Alcon-Giner, Shabhonam Caim, Suparna Mitra, Jennifer Ketskemety, Udo Wegmann, John Wain, Gusztav Belteki, Paul Clarke, Lindsay J. Hall

**Affiliations:** 1 The Gut Health and Food Safety Programme, Quadram Institute Bioscience, Norwich Research Park, Colney, Norwich, UK; 2 Leeds Institute for Biomedical and Clinical Sciences, University of Leeds, Leeds, UK; 3 Norwich Medical School, University of East Anglia, Norwich Research Park, Colney, Norwich, UK; 4 Neonatal Intensive Care Unit, The Rosie Hospital, Cambridge University Hospitals NHS Foundation Trust, Cambridge, UK; 5 Neonatal Intensive Care Unit, Norfolk and Norwich University Hospitals NHS Foundation Trust, Norwich, UK

**Keywords:** Extremely low birth weight infants, Microbiota, 16S rRNA gene sequencing, Shotgun sequencing, DNA extraction, *Bifidobacterium*

## Abstract

**Background:**

Infants born prematurely, particularly extremely low birth weight infants (ELBW) have altered gut microbial communities. Factors such as maternal health, gut immaturity, delivery mode, and antibiotic treatments are associated with microbiota disturbances, and are linked to an increased risk of certain diseases such as necrotising enterocolitis. Therefore, there is a requirement to optimally characterise microbial profiles in this at-risk cohort, via standardisation of methods, particularly for studying the influence of microbiota therapies (e.g. probiotic supplementation) on community profiles and health outcomes. Profiling of faecal samples using the 16S rRNA gene is a cost-efficient method for large-scale clinical studies to gain insights into the gut microbiota and additionally allows characterisation of cohorts were sample quantities are compromised (e.g. ELBW infants). However, DNA extraction method, and the 16S rRNA region targeted can significantly change bacterial community profiles obtained, and so confound comparisons between studies. Thus, we sought to optimise a 16S rRNA profiling protocol to allow standardisation for studying ELBW infant faecal samples, with or without probiotic supplementation.

**Methods:**

Using ELBW faecal samples, we compared three different DNA extraction methods, and subsequently PCR amplified and sequenced three hypervariable regions of the 16S rRNA gene (V1 + V2 + V3), (V4 + V5) and (V6 + V7 + V8), and compared two bioinformatics approaches to analyse results (OTU and paired end). Paired shotgun metagenomics was used as a ‘gold-standard’.

**Results:**

Results indicated a longer bead-beating step was required for optimal bacterial DNA extraction and that sequencing regions (V1 + V2 + V3) and (V6 + V7 + V8) provided the most representative taxonomic profiles, which was confirmed via shotgun analysis. Samples sequenced using the (V4 + V5) region were found to be underrepresented in specific taxa including *Bifidobacterium,* and had altered diversity profiles. Both bioinformatics 16S rRNA pipelines used in this study (OTU and paired end) presented similar taxonomic profiles at genus level.

**Conclusions:**

We determined that DNA extraction from ELBW faecal samples, particularly those infants receiving probiotic supplementation, should include a prolonged beat-beating step. Furthermore, use of the 16S rRNA (V1 + V2 + V3) and (V6 + V7 + V8) regions provides reliable representation of ELBW microbiota profiles, while inclusion of the (V4 + V5) region may not be appropriate for studies where *Bifidobacterium* constitutes a resident microbiota member.

**Electronic supplementary material:**

The online version of this article (10.1186/s12864-017-4229-x) contains supplementary material, which is available to authorized users.

## Background

Infants born less than 37 weeks of gestation are defined as preterm, and account for 1 in 10 live births globally, and a rising proportion of births overall [1]. Notably, complications of preterm birth are the major cause of infant morbidity and mortality; accounting for approximately 1 million deaths worldwide per year. In particular, extremely low birth weight (ELBW) infants (born with a birth weight < 1000 g) have an underdeveloped gut (e.g. including altered pH), and immune system (e.g. reduced expression of microbial pattern recognition receptors) [2]. Furthermore, they are often exposed to external factors that can adversely impact early life gut microbiota colonisation such as Caesarean (C-) section delivery, and frequent antibiotic treatments [3]. The microbiota plays a key role in immune programming [4], pathogen resistance [5], and neurocognitive development [6], and as such microbiota disturbances are linked to negative health outcomes. Notably, ELBW infants have distinct gut microbial communities compared to their full-term counterparts [7]**,** which may directly predispose them to gut bacterial disturbances, and life threatening diseases such as necrotising enterocolitis (NEC) and sepsis [8].

The advent of high-throughput sequencing technologies, has contributed enormously to our understanding of gut microbial diversity in humans, including in term and preterm infants. However, given the challenge of obtaining samples from ELBW infants, this group has received less attention. Nevertheless, the limited sequencing studies performed so far clearly show a higher abundance of *Enterobacteriaceae, Enterococci* and *Staphylococci* and a lower abundance of *Bifidobacteriaceae* and *Lactobacilli* [7]. Importantly *Bifidobacteriaceae* are a dominant member of the full-term infant microbiota (particularly in vaginally delivered breast-fed infants) and are associated with improved host wellbeing [9, 10], and have been used for many years as ‘probiotics’ [11]. Therefore, probiotic supplementation (or microbiota therapy) represents an attractive approach for beneficially manipulating the ELBW gut microbiota in order to improve health outcomes [12].

16S rRNA sequencing is a common, cost-effective, amplicon sequencing method that targets variable regions of the gene encoding the bacterial 16 s rRNA subunit, and can be analysed to determine the bacterial taxa present in a given sample. Notably, previous studies examining the gut microbiota using 16S rRNA gene sequencing in infants have highlighted that the DNA extraction method, and the annealing efficiency of the primers used for the amplification step, can significantly impact the representative bacterial profile obtained [13]. Furthermore, the 16S rRNA hypervariable region (V1 to V9) targeted for sequencing influences the ability to distinguish between different bacterial taxa, and only near-complete 16S rRNA sequences give accurate measures of taxonomic diversity [14]. However, the 16S rRNA gene (~1400 bp) is beyond the read length of current short-read high-throughput sequencing technologies (i.e. Illumina platforms), precluding complete 16S rRNA profiling for high sample volume projects. Thus, it is essential to determine the optimum region that can provide the most representative taxonomic profile for the relevant organisms being investigated.

**Fig. 1:**
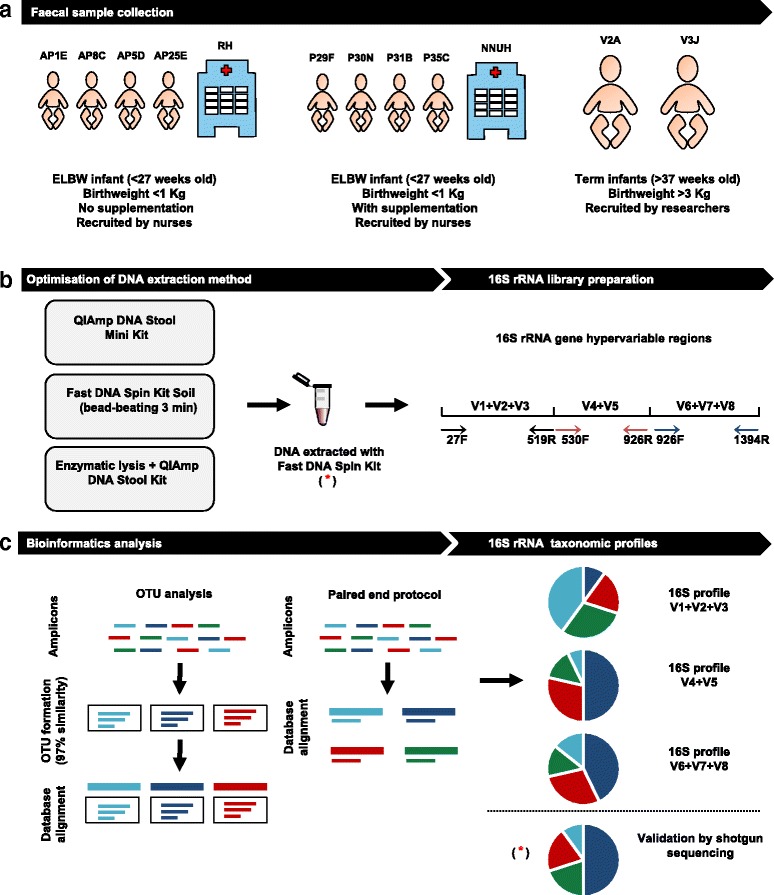
Study pipeline**. a** Recruitment of ELBW infants (<1000 g) with no supplementation (AP1E, AP8C, AP5D and AP25D) and ELBW infants with supplementation (P29F, P30N, P31N, P35C) by nurses at the Rosie Hospital (RH) and the NNUH respectively. Term babies (V3 J, V2A) were recruited by researchers. **b** Optimisation of the bacterial DNA extraction protocol from ELBW infant faeces by testing three different DNA extraction methods (QIAmp DNA Stool Mini Kit, Fast DNA Spin Kit Soil and enzymatic lysis + QIAmp DNA Stool Kit). Bacterial DNA from the study samples was extracted using the Fast DNA Spin Kit Soil and used to prepare three different 16S rRNA gene sequencing libraries. Each library was prepared using a specific pair of primers which target different hypervariable regions (prefixed by a V) of the bacterial 16S rRNA gene: primers 27F-519R target (V1 + V2 + V3), primers 530F-926R target (V4 + V5), and primers 926F-1394R target (V6 + V7 + V8). **c** A preliminary bioinformatics analysis was performed on two samples using two different bioinformatics pipelines: OTU analysis and the PE protocol. Both bioinformatics approaches were used to compare the different 16S rRNA gene sequencing profiles obtained for the different hypervariable regions tested (V1 + V2 + V3, V4 + V5, and V6 + V7 + V8). (*) Validation of the 16S rRNA sequencing results was performed on three samples (AP8C, P29F and V3 J) by shotgun sequencing

In this study, we present an optimised 16S rRNA gene sequencing protocol for obtaining an accurate representation of the gut microbiota composition of at-risk ELBW infants. We emphasise the detection of *Bifidobacterium* and *Lactobacillus*, the bacteria that constituted the probiotic supplementation under investigation. We analysed faecal samples from ELBW preterm infants (with/without *Bifidobacterium* and *Lactobacillus* supplementation) and samples from term infants. Samples from supplemented ELBW infants comprise the ‘spiked’ samples with known species of *Bifidobacterium* and *Lactobacillus*. We optimised a bacterial DNA extraction method for these samples using three different methods, and generated amplicons to three different hypervariable regions of the 16S rRNA gene (V1 + V2 + V3), (V4 + V5) and (V6 + V7 + V8) followed by Illumina sequencing, and analysed the samples using two different bioinformatics pipelines (OTU via QIIME versus paired end protocol (PE), both analysed against the SILVA database). Finally, to validate our analysis further, we performed shotgun sequencing on a subset of the tested samples. Figure 1 shows a summary of the pipelines used in this study. This study demonstrates (i) the requirement for an extended bead-beating step during DNA extraction from faecal samples, and (ii) sequencing regions (V1 + V2 + V3) and (V6 + V7 + V8) of the 16S rRNA gene provide the most representative bacterial profile of the ELBW infant gut microbiota.

## Methods

### Subject recruitment and faecal sample collection

This study was approved by the University of East Anglia (UEA) Faculty of Medical and Health Sciences Ethics Committee, and sample collection was in accordance with protocols laid out by the National Research Ethics Service (NRES) approved UEA Biorepository (Licence no: 11,208). Infants admitted to the Neonatal Intensive Care Units (NICUs) of the Norfolk and Norwich University Hospital (NNUH, Norwich, UK) and the Rosie Hospital (Cambridge, UK) were recruited by doctors or nurses with informed and written consent obtained from parents. Both NICUs had similar protocols for feeding and the prescription of antibiotics and antifungal drugs. The Rosie Hospital does not use probiotics, whilst the NNUH routinely prescribed all ELBW infants an oral probiotic treatment containing *Bifidobacterium bifidum* and *Lactobacillus acidophilus* (Infloran®, Desma Healthcare, Switzerland) in a twice daily dose of 1 × 10^9^ of each species, given from birth until 34 weeks old (these also represent ‘spiked’ ELBW infants with known bacteria, thus useful for downstream analysis). We recruited a total of eight ELBW infants, four received probiotic supplementation and four did not receive any supplementation. All recruited ELBW infants were <27 week’s gestation and weighed ≤1000 g at birth. We specifically selected infants born vaginally and breast-fed, with the aim of normalising for other external factors, which can influence gut colonisation of *Bifidobacterium and Lactobacillus*. A control group of two term babies were also recruited by the research team following the same protocol. Faecal samples were collected from nappies into a sterile stool container and stored at 4 °C. DNA was extracted within 4 h of collection. Subject details are included in Additional file 1: Table S1.

### Sample processing and DNA extraction

Optimisation of bacterial DNA extraction was performed on faecal samples from two ELBW infants (with/without supplementation) and one term infant sample.

Three different DNA extraction methods were used: (i) FastDNA Spin Kit for Soil (MP) following the manufacturer’s instructions and extending the bead-beating step to 3 min (ii) QIAmp DNA Stool Mini Kit (Qiagen) following the manufacturer’s instructions, and (iii) QIAmp DNA Stool Mini Kit (Qiagen) including an initial enzymatic lysis step of 1 h at 37 °C (enzymatic mix: 50 mM Tris-HCl, pH 8.0, 10 mM MgSO_4_, 5 mg/mL lysozyme and 50 U/mL mutanolysin). The DNA recovered from these samples was assessed using a Qubit® 2.0 fluorometer (Invitrogen).

### 16S rRNA gene library preparation

Fast DNA Spin Kit extracted DNA was used for preparing 16S rRNA Illumina MiSeq sequencing libraries. DNA concentration was normalised to 5 ng/mL using a Qubit® 2.0 fluorometer. Three hypervariable regions of the 16S rRNA gene (V1 + V2 + V3 (primers 27F-519R), V4 + V5 (primers 530F-926R), and V6 + V7 + V8 (primers 926F-1394R)) were amplified using the HotStarTaq Plus Master Mix Kit (Qiagen, USA). Details of the primer sequences used for amplification can be found in Additional file 2: Table S2. Each DNA sample was amplified using a primer pair tagged individually with a unique barcode. PCR amplification conditions were: 1 cycle of 94 °C for 3 min, followed by 25 cycles of 94 °C for 45 s, 55 °C for 15 s and 72 °C for 30 s. Amplicons were pooled in equal proportions and purified using Ampure XP beads (Agencourt). The purified product was used to prepare the Illumina DNA library. Libraries were sequenced on the Illumina MiSeq platform using a read length up to 2 × 300 bp.

### Whole genome shotgun metagenomics library preparation

Genomic DNA (approximately 500 ng) from two ELBW infant samples (with/without supplementation), and one term infant sample was fragmented to an average size of 250 bp and subjected to DNA library creation using established Illumina paired end protocols. Adapter-ligated libraries were amplified and indexed via PCR. A portion of each library was used to create an equimolar pool and enriched libraries were subjected to 100 base paired end sequencing (HiSeq 2000 V3; Illumina).

### Bioinformatics analysis

#### 16S rRNA gene sequencing analysis

Two bioinformatics pipelines were used to analyse the 16S rRNA gene sequencing data: OTU clustering analysis and paired end protocol (PE). OTU clustering analysis was performed using the QIIME bioinformatics pipeline [15]. First, read pairs were assembled using PEAR [16], a highly accurate pair-end read merger. Second, sequences were quality filtered using QIIME’s split_libraries_fastq.py and chimeras were identified and removed using identify_chimeric_seqs.py and filter_fasta.py respectively. Following, OTU picking step was run using pick_open_reference_otus.py (percent_subsample parameter set at 0.1) and QIIME SILVA_128 [17] as our reference database. OTUs were formed by clustering to 97% similarity, and a representative sequence was picked for each OTU aligned using PyNAST [18] and taxonomy was assigned using uclust [19]. Filtering prior to build the tree that was done by removing the positions with gaps and specified as 0 in the lanemask. FastTree is used to create a tree file for the represented sequences. Final taxonomic output was saved as a biom file. More details of the scripts used to run the QIIME pipeline can be found in Additional file 3.

We also used an in-house PE protocol following the quality control of the raw paired reads using FASTX-Toolkit [17] (with a minimum quality threshold of 33 for at least 50% of the bases in each read sequence. Reads that passed the threshold were aligned against the SILVA database (version: SILVA_128_SSURef_tax_silva) [20] and BLASTN (ncbi-blast-2.2.25+; Max e-value 10e-3) [21]. We then imported the BLAST files on MEGAN6 [22] to create MEGAN-own files (“rma6” files) using the following parameters: 100 as maximum number of matches per reads, and “Min Score = 50” and “Top Percent = 10”. All output files (rma6) of paired read sequences were then normalised and compared using MEGAN6.

#### Whole genome shotgun gene sequencing

Whole genome paired sequences from samples AP8C (an ELBW infant without supplementation), P29F (an ELBW infant who received supplementation) and V3 J (term infant) were obtained from an Illumina HiSeq 2000 V3 sequencer. The first 10 bases were trimmed using FASTX-Toolkit [23]. Subsequently, trimmed sequences were aligned against the NCBI non-redundant database (04/2016) [24] using DIAMOND [25]. All output files of paired read sequences were then imported and analysed using the PE protocol of MEGAN with non-default settings.

Functional profiles were performed on the same samples using the KEGG pathway database. Mapping files used for this analysis were obtained from MEGAN’s website.

### Sequencing reads statistics

Read counts at different stages of the bioinformatics analysis are provided in Additional file 4. To compare study samples, sequences were normalised using values from the sample with the lowest number of reads. In other cases, read counts were displayed in percentage of number of reads.

Principal Coordinate Analysis plot was performed using Bray-Curtis distances on the 16S rRNA bacterial community profiles using MEGAN. The Shannon diversity index was obtained by exporting genus level profile (normalised) from all 30 samples in MEGAN and plotting them in Excel.

### Primer annealing study

Amplicon sequences from the most common bacterial taxa found in sample P29F (ELBW infant with supplementation) were extracted using MEGAN [22]. Full length sequences of the respective 16S rRNA genes were obtained from Genbank after identified the respective database entries using BLASTN [26]. Primer annotation of the 16S rRNA sequences was performed using Genedoc 2.7.

### Validation of primers 530F-926R: PCR and melting curves qPCR

#### PCR

DNA extracted from *B. bifidum* (isolated from the probiotic supplement) and seven different *Bifidobacterium* strains (from NCIMB strain collection, Aberdeen, Scotland), was amplified by PCR using primers 530F-926R. Additional file 5: Table S3 provides the details of the NCIMB collection strains used in this study. A faecal metagenomic sample and a *Lactobacillus acidophilus* strain (isolated directly from the probiotic supplement) were used as positive controls. Amplicon samples were run on 1% agarose gel for 30 min at 100 V. DNA was visualized under UV light after staining with ethidium bromide.

#### qPCR

Melting curves of PCR amplicons obtained from the probiotic strains (*Bifidobacterium bifidum* and *Lactobacillus acidophilus*), and two bacterial isolates from an ELBW infant with supplementation (*Enterococcus faecium* and *Streptococcus infantarius*) were performed using a LightCycler 480 (Roche Molecular Diagnostics). Conditions for the melting curves were: 95 °C for 5 s, 65 °C for 1 min and a final stage at 97 °C continuous. As an additional experiment, a melting curve from an amplicon obtained from a mixed DNA sample (containing 5 ng DNA from of each of the above bacterial species) was run. Conditions used for this melting curve were the same as the ones described previously.

## Results and discussion

### Effect of DNA extraction method in sample preparation.

Sample collection and DNA extraction are the first critical steps for microbiota NGS studies [27]. Previous studies have indicated that refrigeration of faecal samples does not significantly influence overall microbiota composition within the first 72 h upon sample collection [28], thus in this study our samples were stored at 4 °C at the hospital sites, before rapid (within 4 h) DNA extraction. This also avoided repeated freeze thawing, which may increase the ratio of *Firmicutes* to *Bacteroidetes* when using PCR based methods (e.g 16S rRNA gene sequencing) for downstream analysis [29].

We then compared three DNA extraction protocols involving two different kits to determine the most appropriate method for extracting bacterial DNA using two faecal samples from two ELBW infants, and one term infant sample. The DNA recovery optimisation procedure indicates that the Fast DNA Spin Kit for Soil was the most effective method at extracting bacterial DNA from ELBW infant faeces (Additional file 6: Table S4). Importantly, this methodology included a bead-beating step, which has been previously shown to improve quality and quantity of the isolate DNA potentially via disruption of cell membrane components (including cells walls and capsules) [13, 30], and this finding has now been expanded to ELBW faecal samples. Furthermore, this method obtained higher DNA yields from all samples, particularly *Bifidobacterium*-supplemented ELBW and *Bifidobacterium*-rich term infants when extending the bead-beating time to 3 min. Indeed, it was only with this DNA extraction protocol that we obtained enough DNA from all samples for subsequent sample sequencing, all other methods provided inadequate quantities, and could therefore not be utilised further. This highlights that samples expected to have high *Bifidobacterium* levels (genus known to express exopolysaccharide capsules) [31] are optimally processed using an extended bead-beating DNA extraction protocol.

### 16S rRNA gene library preparation and bioinformatics analyses (OTU pipeline versus PE protocol)

Previous studies have indicated that targeting different variable 16S rRNA regions can influence the microbiota profiles obtained. Therefore, to determine which regions are the best target for ELBW infant samples, we next prepared three 16S rRNA gene sequencing libraries by amplifying different regions of the 16S rRNA gene (V1 + V2 + V3, V4 + V5, and V6 + V7 + V8). To perform a thorough analysis of the data, we initially completed a preliminary study on one ELBW infant (AP1E) and one term infant sample (V3 J) using two different bioinformatics approaches; (i) a reference-based OTU clustering analysis employing the open-source pipeline QIIME, which clusters the raw reads into OTUs of 97% similarity before aligning them against the database, and (ii) the PE protocol, which directly blasts the raw reads to the database after quality control. It is important to highlight that this preliminary study was performed using the same database (SILVA version 128) for both bioinformatics approaches, to minimise any differences in relation to mapping reads.

**Fig. 2:**
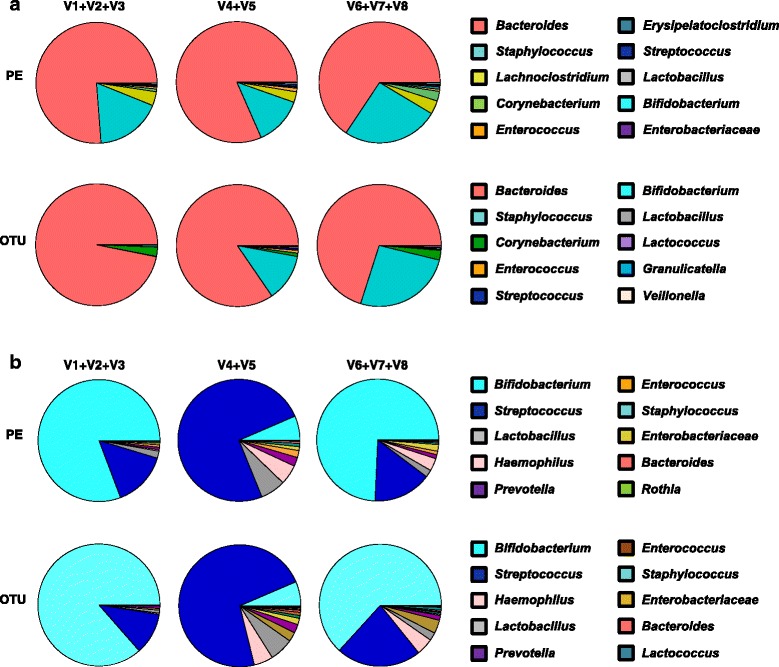
Comparison of bioinformatics analyses (OTU versus PE protocol). Preliminary study comparing two different bioinformatics approaches: OTU clustering performed using QIIME and PE protocol. Both bioinformatics approaches used the same database (SILVA version 128). **a** Taxonomic profiles obtained using PE protocol and OTU clustering for sample AP1E (ELBW infant no supplementation). **b** Taxonomic profiles obtained using PE protocol and OTU clustering for sample V3 J (term infant sample). Three different 16S rRNA gene libraries were prepared for each sample, V1 + V2 + V3, primers 27F-519R, V4 + V5, primers 530F-926R and V6 + V7 + V8, primers 926F-1394R. Further information on the number of reads obtained for this study can be found in Additional file 8

Results from this comparative study showed that both methods tested (OTU vs PE) presented similar taxonomic profiles at genus level for the majority of the bacterial genera detected (Fig. 2). Indeed, as faecal samples from ELBW infants generally exhibit low bacterial diversity (therefore excellent sequencing coverage), this may indicate why both bioinformatics pipelines show similar trends, although this may be somewhat different if a more complex (e.g. adult) sample was compared. Interestingly, for bacterial genera present in low numbers, such as *Lactobacillus* or *Haemophilus,* the results indicate small differences between the pipelines. This may be explained by the fact that the QIIME uses a custom database that only contains specific marker sequences [32]. Thus, we would not expect to map all reads using QIIME, which may result in lower sensitivity, whereas the PE protocol takes all quality filtered reads into account and discards low-confidence taxa (assignments <25 reads). In this comparative study, we also calculated the Shannon Diversity Index (Additional file 7: Figure S1), which indicated that both approaches (OTU and PE) were comparable with the exception for region V1 + V2 + V3 (27F-519R), which presented the lowest value when using the OTU approach.

Thus, comparison of OTU vs. PE comparison bioinformatics pipelines, using the same database, indicates similar profiles obtained in the most abundant taxa, whereas potentially different and distinct bacterial profiles for those genera present in lower abundance. Additional file 8 provides the details of the number of reads obtained by the OTU analysis and the PE protocol.

### Effect of 16S rRNA gene hypervariable region amplified and taxonomic assignments

Initially we assessed coverage of our sequence data by performing rarefaction curves. Our analysis indicated that at 25,000 reads the vast majority of bacterial populations were sequenced, thus at this depth we captured sample diversity. This enabled us to normalise our data across samples for subsequent comparisons, which was important since we observed that there were some differences between the read counts, in particular region (V4 + V5) generated between 5 and 10 times the number of reads when compared to regions (V1 + V2 + V3, and V6 + V7 + V8) (Additional file 9: Figure S2).

**Fig. 3:**
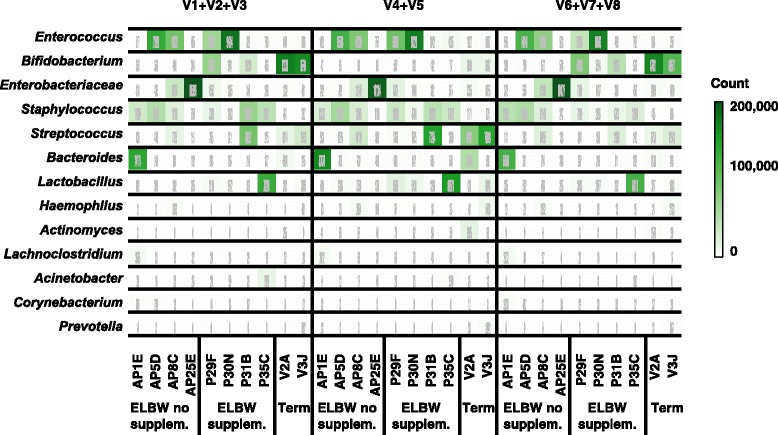
Comparison of taxonomic assignments among the 16S rRNA gene hypervariable regions tested using PE protocol approach. Heat map displaying number of reads assigned to the most common bacterial taxa found in the study samples. Top panel row divides the figure in the different regions of the 16 s rRNA gene analysed, namely: V1 + V2 + V3 (primers 27F-519R), V4 + V5 (primers 530F-926R) and V6 + V7 + V8 (primers 926F-1394R). The vertical axis of the panel indicates a selection of the 13 most common bacterial taxa found. The horizontal axis labels the different samples used in the study: preterms without supplementation (AP1E, AP5D, AP8C, AP25C), preterms with supplementation (P29F, P30N, P31B, P35C), and term baby samples (V2A, V3 J). The intensity of the green colour highlights the abundance of the number of reads found. Probiotic supplementation has been abbreviated to supplem. in the figure. Further information on the number of reads obtained for this study can be found in Additional file 19

When comparing the taxonomic assignments obtained from the different 16S rRNA libraries amplifying three hypervariable regions (V1 + V2 + V3, V4 + V5, and V6 + V7 + V8), we observed that data from the most abundant bacterial genera found in ELBW samples (e.g. *Enterococcus, Staphylococcus,* and *Streptococcus*) were similar (Fig. 3). These bacterial genera have all previously been described as common members of the gut microbiota of preterm infants [33], and results obtained in this study also indicate that the three hypervariable regions similarly target these bacterial taxa.

As we have ‘spiked’ or supplemented ELBW infants, we can effectively use the known bacterial taxa (*Lactobacillus* and *Bifidobacterium*) as control populations. Notably, when we examined the number of reads assigned to these genera, the results indicated significant dissimilarities between hypervariable regions.

In the case of *Lactobacillus*, the three hypervariable regions (V1 + V2 + V3, V4 + V5, and V6 + V7 + V8) were able to detect the presence of this genus at significant levels (>1000 reads) in three (P29F, P30N and P35C) out of the four samples from ELBW infants who received supplementation and in one (un-supplemented) term baby sample (V3 J). Amplicons from region (V4 + V5) revealed 3 and 6 times higher number of reads for *Lactobacillus* when compared to the other regions (V1 + V2 + V3 and V6 + V7 + V8). These data indicate that region (V4 + V5) may over-represent this bacterial genus, which is validated and discussed in more detail in a later paragraph when comparing to shotgun analysis.

The taxonomic assignments obtained for *Bifidobacterium* reveal prominent differences between the different regions. Analysis of region (V4 + V5) did not detect *Bifidobacterium* at high levels (>1000 reads) in any of the four samples (P29F, P30N, P31B and P35C) tested from ELBW infants who received supplementation (i.e. ‘spiked’ samples). In contrast, the other regions (V1 + V2 + V3 and V6 + V7 + V8) did show *Bifidobacterium* at >1000 reads assigned in three out of the four samples (P29F, P31B, P30N) analysed from ELBW infants who had received probiotic supplementation (Fig. 3). Importantly, the remaining supplemented ELBW infant (P35C) had recently finished a 5-day course of vancomycin, which could explain the underrepresentation of *Bifidobacterium* in this sample. Furthermore, the results from region (V4 + V5) in samples from term babies (which normally contain a higher amount of *Bifidobacterium* than preterm babies) followed the same trend as the ELBW infants tested, revealing a 93% decrease in the number of reads assigned to *Bifidobacterium* compared to the other regions (V1 + V2 + V3 and V6 + V7 + V8). This underrepresentation of *Bifidobacterium* agrees with previous studies that also highlighted problems with amplifying the (V4 + V5) region of the 16S rRNA gene from faecal samples of adults and infants [34, 35]. We also performed the same analysis using the QIIME pipeline (using the same database as the PE protocol). Interestingly, analysis via QIIME produced very similar findings; overrepresentation of *Lactobacillus* and underrepresentation of *Bifidobacterium* when using region V4 + V5 (Additional file 10: Figure S4).

**Fig. 4:**
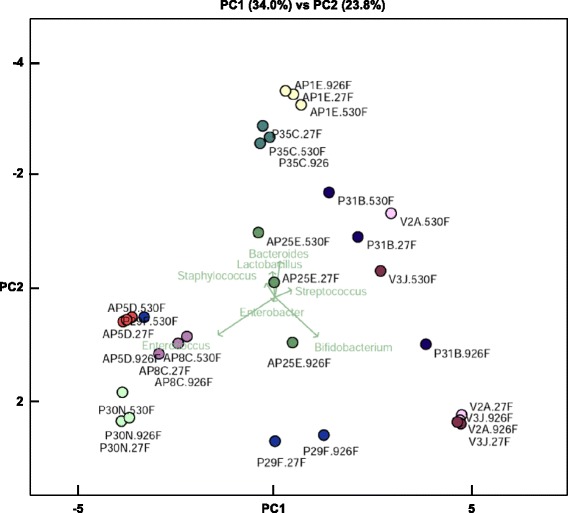
Principal Coordinate Analysis (PCoA) based on 16S rRNA community profiles analysed using PE protocol of the hypervariable regions tested. PCoA was performed based on the taxonomic assignments obtained from the 16S rRNA gene sequencing libraries analysed. Samples used for this plot were classified in main three groups: preterms without supplementation (AP1E, AP5D, AP8C, AP25C), preterms with supplementation (P29F, P30N, P31B, P35C), and term baby samples (V2A, V3 J). Samples names are coded highlighting the 16S rRNA gene library they belong. Sample names ending in (.27F) belong to 16S rRNA gene library prepared using primers 27F-519R (target region V1 + V2 + V3), sample names ending in (.530F) belong to 16S rRNA gene library prepared using primers 530F-926R (region V4 + V5), and sample names ending in (.926F) belong to 16S rRNA gene library amplified using primers 926F-1394R (region V6 + V7 + V8). PCoA plot indicates that distribution of samples targeting (V4 + V5) region was distinct from samples targeting (V1 + V2 + V3) and (V6 + V7 + V8)

Notably, when we performed a Principal Coordinate Analysis (PCoA) based on 16S rRNA community profiles of the hypervariable regions tested (Fig. 4), the distribution of samples amplified using region (V4 + V5) was distinct from samples amplified using region (V1 + V2 + V3 and V6 + V7 + V8). These differences were more accentuated in faecal samples which contained *Bifidobacterium* such as P31B and P29F (from supplemented ELBW infants) and V3 J and V2A (from un-supplemented term infants). The PCoA plot performed using the QIIME bioinformatics pipeline showed the same findings (Additional file 11: Figure S5).

Furthermore, we also performed Shannon diversity analysis on all samples (Additional file 12: Figure S3), which indicated that region V4 + V5 appeared to have higher diversity, when compared to the other regions particularly for *Bifidobacterium*-rich samples (V3 J and V2A). Although sample number is limited, it should be noted that targeting different regions of 16S rRNA may lead to different diversity interpretations.

### Primer annealing study and validation of primers 530F-926R (region V4 + V5) against *Bifidobacterium*: PCR and melting curve analysis

To investigate any possible primer annealing problems, we aligned the sequences of the three primer pairs used to construct the 16S rRNA libraries to 16S rRNA gene sequence from the probiotic strain *Bifidobacterium bifidum*, and other bacterial members commonly found in the samples from the ELBW infants. Surprisingly, primers amplifying region (V4 + V5, 530F-926R) did not reveal any obvious annealing disadvantage (mismatch) towards *Bifidobacterium* (Additional file 13: Figure S6), while primers amplifying region (V1 + V2 + V3, 27F) and region (V6 + V7 + V8, 926F) presented mismatches (previously highlighted in other studies (13)), against the *Bifidobacterium* strains tested. The *in-silico* analysis was complemented by direct amplification of the 16S rRNA (V4 + V5) region, using genomic DNA isolated from seven different strains of *Bifidobacterium* including the probiotic strain *B. bifidum* (Additional file 14: Figure S7). This experiment confirmed that the primer pair 530F-926R did not encounter any annealing problem when working with DNA isolated from pure strains, which is in agreement with our annealing study results.

Further investigation focused on the GC content of region (V4 + V5) of the strains used in the probiotic supplementation *(B. bifidum* and *L. acidophilus*) and two other strains which were overrepresented by this region, *Enterococcus faecium* and *Streptococcus infantarius*. Several studies have described that templates with a high GC content (e.g. *Bifidobacterium,* as confirmed in Additional file 15: Figure S8a) are more difficult to amplify than non-GC-rich templates [36, 37]. In the context of a metagenomic sample, where different genomes are competing against the same pair of primers, differences in GC content would be expected to significantly impact amplification, and thus downstream analysis. Notably, using the same PCR conditions, but in this instance using mixed template DNA (i.e. combined genomic DNA from all strains (*B. bifidum, L. acidophilus, E. faecium and S. infantarius*), to simulate a mixed community sample, primers 530F-926R preferentially amplified the region (V4 + V5) of other bacterial genomes (confirmed by presence of peak 1 in Additional file 15: Figure S8b) before *B. bifidum* (represented by peak 2 in the same figure). Therefore, these data suggest that the higher GC content of *Bifidobacterium* in region (V4 + V5) may lead to an underrepresentation of *Bifidobacterium* when it is present in a metagenomic sample. Other studies using the same region (V4 + V5), but different primers, have also encountered an underrepresentation of *Bifidobacterium* [34]. It is also interesting to highlight that primer 926R presented the lowest GC content among the primers used in this study and it is the only one which does not have a GC clamp at its 3’end, which could also interfere with binding to genomes with high GC content.

### Validation of 16S rRNA gene sequencing data using shotgun metagenomic analysis

To validate the 16S rRNA gene sequencing results, we performed shotgun sequencing on paired DNA samples from infants AP8C, P29F and V3 J. This technology introduces less PCR bias and artefacts, but is significantly more expensive to scale up and requires additional computing power for downstream analysis, which in large-scale in vivo and clinical studies are important factors to consider. From a sample collection stand-point, shotgun metagenomic sequencing also requires a higher yield of bacterial DNA (500 ng is the recommended amount of DNA compared to 25 ng required for 16S rRNA gene sequencing), which can be challenging to obtain from case-specific ELBW infants (e.g. after prophylactic antibiotic administration).

**Fig. 5:**
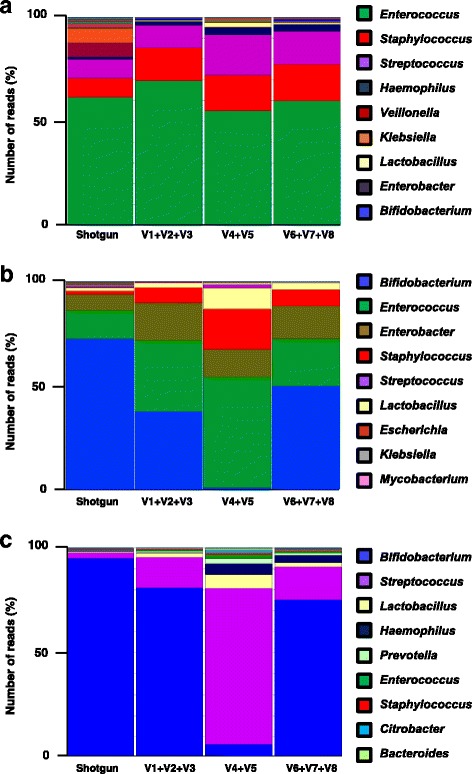
Bacterial community profiles determined by shotgun and 16S rRNA gene sequencing data. Comparison of bacterial profiles analysed by shotgun and 16S rRNA gene sequencing data. Normalised data and relative abundance of the bacterial taxa was represented in percentages of number of reads. Bar colours represent different genus taxa, and bar lengths signify the relative abundance of each taxon. 16S rRNA bacterial profiles are named according to the different 16S rRNA hypervariable region amplified: (V1 + V2 + V3, primers 27F-519R), (V4 + V5, primers 530F-926R), and (V6 + V7 + V8, primers 926F-1394R). **a** Bacterial community profiles determined by shotgun and 16S rRNA gene sequencing from an ELBW infant (sample AP8C) with no supplementation. **b** Bacterial community profiles determined by shotgun and 16S rRNA gene sequencing from an ELBW infant (sample P29F) with supplementation. **c** Bacterial community profiles determined by shotgun and 16S rRNA gene sequencing from a term baby (sample V3 J). More detailed information on the number of reads obtained by shotgun and 16S rRNA gene sequencing data can be found in Additional file 20

**Fig. 6:**
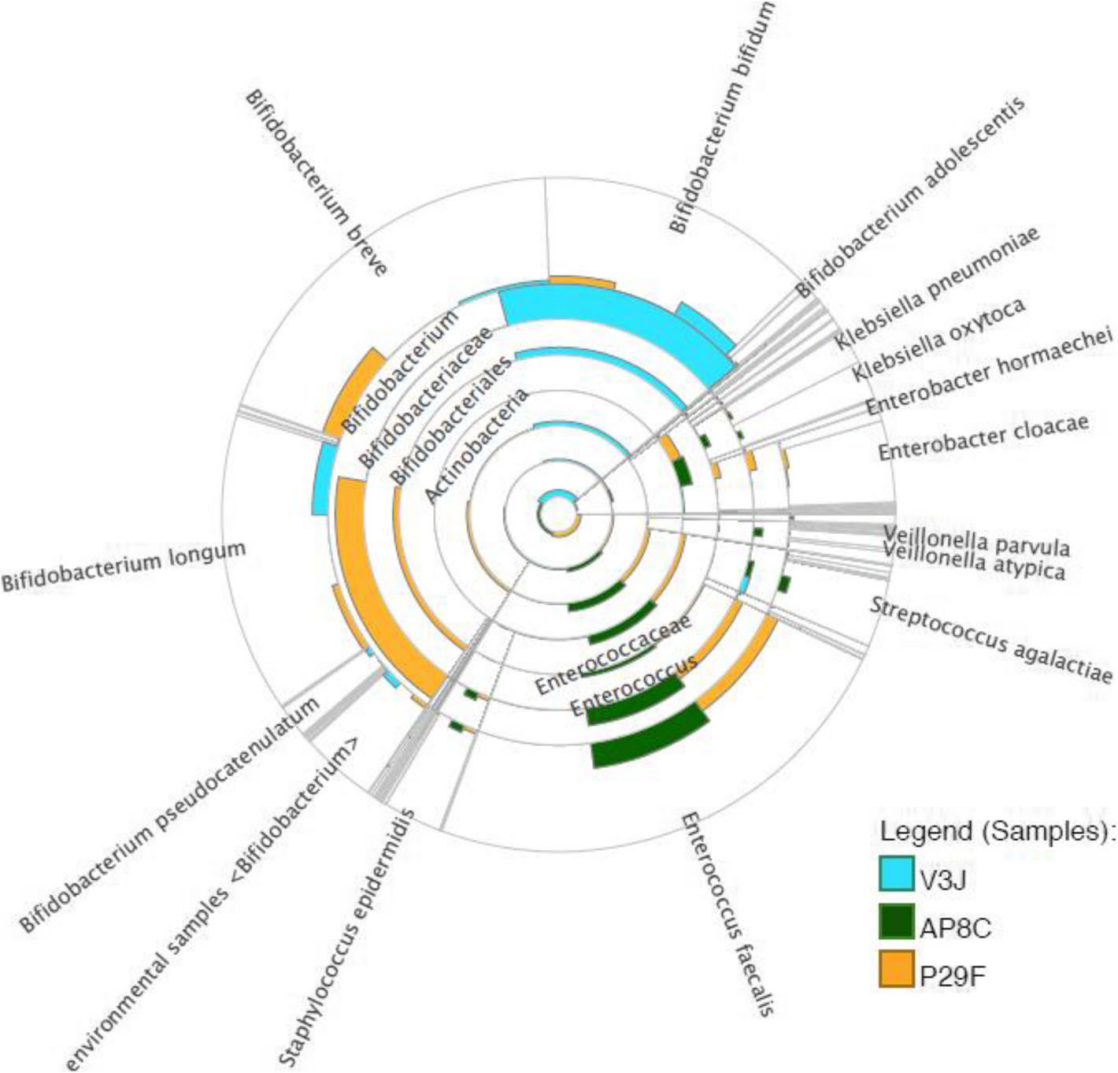
Shotgun taxonomic profiles from two ELBW infants with/without supplementation and a term infant. Radial taxonomic tree displaying shotgun community profiles from faecal samples of an ELBW infant with no supplementation (AP8C, represented in green) an ELBW infant with supplementation (P29F, represented in yellow) and a term baby (V3 J, represented in blue). Relative abundance was indicated according to the length of the coloured bars in the figure. The centre of the radial tree indicates phylum level, and the subsequent concentric layers of the radial tree indicate class, order, family, and genus and species level. Term baby (V3 J) and ELBW infant with supplementation (P29F) samples presented a higher abundance of *Bifidobacterium* when compared to an ELBW infant with no supplementation (AP8C)

Results from whole genome shotgun sequencing confirmed the presence of the most predominant bacterial genera detected using 16S rRNA gene sequencing, namely *Bifidobacterium, Enterococcus, Staphylococcus, Enterobacter* and *Streptococcus* using PE protocol (Fig. 5) and QIIME pipeline (Additional file 16: Figure S9). The additional coverage that this method provided when compared to 16S rRNA sequencing data, also enabled us to confirm the presence of *Bifidobacterium* in sample P29F (ELBW infant with supplementation), and more specifically the presence of *Bifidobacterium bifidum* (Fig. 6), which corresponds to the species present in the supplementation given to these infants. This result may also correlate with functional analysis (Additional file 17: Figure S10), which indicated an increase in glycan metabolism pathways (in sample P29F), as *B. bifidum* has been previously been shown to metabolise breast milk-derived human milk oligosaccharides [38].

When we compare the taxonomic assignments from metagenomic shotgun sequencing to the taxonomic assignments from 16S RNA gene profiling of the three hypervariable regions we found that (V4 + V5) region failed to adequately discriminate the gut bacterial population from ELBW infants. This region overrepresented *Streptococcus, Enterococcus, Staphylococus* and *Lactobacillus* genera, and underrepresented *Bifidobacterium* in comparison to the other regions. The percentages of the number of reads obtained are indicated in Table 1.

**Table 1:**
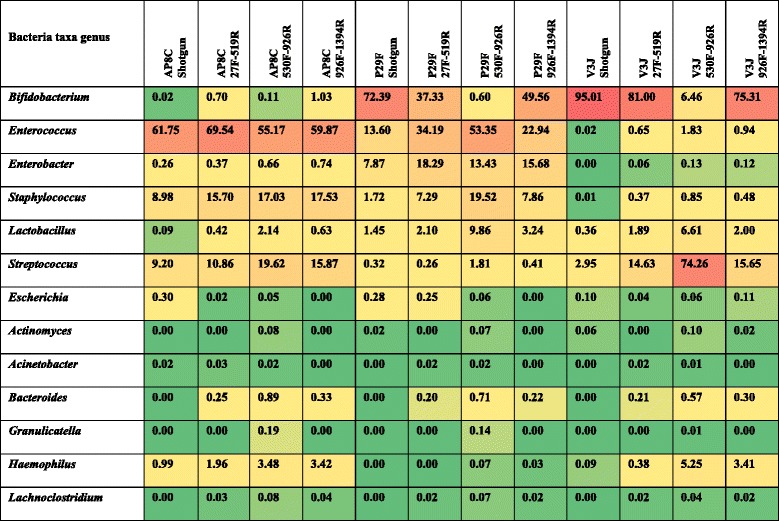
Percentage of number of reads obtained for shotgun and 16S rRNA gene sequencing. Data represented in this table corresponds to three different study groups: ELBW infant with no supplementation (AP8C), ELBW infant with supplementation (P29F) and a term infant (V3 J). Bacterial taxa column indicates the twelve most common bacteria present (further details can be found in Additional file 20)

To visualise if there are strong patterns between the 16S rRNA bacterial profiles of the different hypervariable regions tested (V1 + V2 + V3, V4 + V5 and V6 + V7 + V8) and shotgun sequencing data (used as gold standard) we performed a PCoA (Additional file 18: Figure S11). The PCoA (using QIIME and PE pipelines) confirmed that region (V4 + V5) amplicons do not cluster with the other 16S rRNA regions (i.e. V1 + V2 + V3 and V6 + V7 + V8) and corresponding shotgun data, with differences further amplified among samples where *Bifidobacterium* is a resident member of the gut microbiota (e.g. differences were greater in sample P29F belonging to an ELBW with probiotic supplementation and sample V3 J from a term infant sample).

## Conclusions

This study highlights the importance of using an optimal DNA extraction method (i.e. including an extended beat-beating step) for 16S rRNA microbiota profiling, which is now considered gold standard by many research teams.

Appropriate primer selection when using 16S rRNA microbiota profiling is essential for analysing gut metagenomic samples. Our study using two bioinformatics approaches (OTU and PE) shows that (V4 + V5) region failed to represent the most common bacterial populations present in the ELBW infant gut microbiome. This region overrepresented *Streptococcus, Enterococcus, Staphylococus* and *Lactobacillus* genera, and underrepresented *Bifidobacterium* when compared to the other hypervariable regions (V1 + V2 + V3 and V6 + V7 + V8). We demonstrated that there is a difference in the GC content of the (V4 + V5) region of the 16S rRNA gene between the latter bacterial genera and *Bifidobacterium,* and our data indicate this may negatively impact *Bifidobacterium* DNA amplification in metagenomic samples. Therefore, we conclude that the V4 + V5 region should be avoided in metagenomics studies that may contain the beneficial genus *Bifidobacterium*, or indeed other taxa with a high GC content.

The 16S rRNA gene sequencing protocol presented in this study will contribute to our understanding of how early life clinical interventions on the gut microbiota of ELBW infants such a microbiota supplementation/therapy, dietary modification, or antibiotics regimens impact the wider microbiota and link to health outcomes.

## Additional files

Additional file 1: Table S13. Subject details and metadata. (PDF 202 kb)

Additional file 2: Table S2. Primers used in 16S rRNA sequencing library. (PDF 137 kb)

Additional file 3: Scripts used to run the QIIME pipeline. (PDF 80 kb)

Additional file 4: Number of raw reads counts for shotgun and 16S rRNA gene sequencing data (PDF 112 kb)

Additional file 5: Table S3. *Bifidobacterium* strains used for validating primers 530F-926R using PCR (PDF 198 kb)

Additional file 6: Table S4. DNA yield from different DNA extraction methods. (PDF 306 kb)

Additional file 7: Figure S1. Shannon diversity index on 16S rRNA gene sequencing data analysed using OTU and PE protocol. Shannon diversity index was calculated using 16S rRNA bacterial community profiles for sample AP1E (ELBW infant without probiotic supplementation) and sample V3 J (term infant). **a** Shannon diversity indexes comparison of three different 16S rRNA libraries (27F-519R (region V1 + V2 + V3), 530F-926R (region V4 + V5) and 926F-1394R (region V6 + V7 + V8)) using OTU and PE protocol pipelines for sample AP1E (preterm no supplementation). **b** Shannon diversity indexes comparison of three different 16S rRNA libraries (27F-519R (region V1 + V2 + V3), 530F-926R (region V4 + V5) and 926F-1394R (region V6 + V7 + V8)) using OTU and PE protocol pipelines for sample V3 J (term infant). (PDF 74 kb)

Additional file 8: Number of reads obtained by PE and QIIME for samples V3 J and AP1E. (PDF 76 kb)

Additional file 9: Figure S2. Rarefaction curves 16S rRNA gene sequencing data. Rarefaction curves representing number of species (leaves) detected at genus level versus number of reads sampled. Three different 16S rRNA gene sequencing data were used for this study: i) green curves represent sequencing data from 16S rRNA library prepared using primers 27F-519R, ii) red curves represent sequencing data from 16S rRNA library prepared using primers 530F-926R and iii) blue curves represent sequencing data from 16S rRNA library prepared using primers 926F-1394R. Rarefaction curves are labelled with numbers to differentiate among the samples used in the study: 1 (V2A.530F), 2 (V2A.926F), 3 (V3 J.926F), 4 (AP8C.530F), 5 (V2AJ.27F), 6 (AP5D.530F), 7 (P35C.530F), 8 (V3 J.530F), 9 (P29F.530F), 10 (P31B.530F), 11 (P30N.530F), 12 (AP25E.530F), 13 (AP25E.926F), 14 (AP25E. 27F), 15 (AP8C.926F), 16 (P35C.926F), 17 (V3 J.27F), 18 (P31B.926F), 19 (AP1E.530F), 20 (P31B.27F), 21(P29F.27F), 22 (P30N.27F), 23 (AP5D.926F), 24 (AP5D.27F), 25 (P29F.926F), 26 (AP1E.926F),27 (AP1E.27F), 28 (AP8C.27F), 29 (P30N.926F), 30 (P35C.27F). Numbers 12, 13 and 14 correspond to sample AP25E where majority of sequenced reads assigned at family level (i.e. *Enterobacteriaceae*). (PDF 62 kb)

Additional file 10: Figure S4. Comparison of taxonomic assignments among the 16S rRNA gene hypervariable regions tested using QIIME approach. Heat map displaying number of reads assigned to the most common bacterial taxa found in the study samples using QIIME bioinformatics pipeline. Top panel row divides the figure in the different regions of the 16 s rRNA gene analysed, namely: V1 + V2 + V3 (primers 27F-519R), V4 + V5 (primers 530F-926R) and V6 + V7 + V8 (primers 926F-1394R). The vertical axis of the panel indicates a selection of the 13 most common bacterial taxa found. The horizontal axis labels the different samples used in the study: preterms without supplementation (AP1E, AP5D, AP8C, AP25C), preterms with supplementation (P29F, P30N, P31B, P35C), and term baby samples (V2A, V3 J). The intensity of the green colour highlights the abundance of the number of reads found. Probiotic supplementation has been abbreviated to supplem. in the figure. Further information on the number of reads obtained for this study can be found in Additional file 19. (PDF 262 kb)

Additional file 11: Figure S5. Principal Coordinate Analysis (PCoA) based on 16S rRNA community profiles analysed using QIIME of the hypervariable regions tested. PCoA was performed based on the taxonomic assignments obtained from the 16S rRNA gene sequencing libraries analysed. Samples used for this plot were classified in main three groups: (i) preterms without supplementation (AP1E, AP5D, AP8C, AP25C), (ii) preterms with supplementation (P29F, P30N, P31B, P35C), and (iii) term baby samples (V2A, V3 J). Samples names are coded highlighting the 16S rRNA gene library they belong. Sample names ending in (.27F) belong to 16S rRNA gene library prepared using using primers 27F-519R (target region V1 + V2 + V3), sample names ending in (.530F) belong to 16S rRNA gene library prepared using primers 530F-926R (region V4 + V5), and sample names ending in (.926F) belong to 16S rRNA gene library amplified using primers 926F-1394R (region V6 + V7 + V8). PCoA plot indicates that distribution of samples targeting (V4 + V5) region was distinct from samples targeting (V1 + V2 + V3) and (V6 + V7 + V8). (PDF 37 kb)

Additional file 12: Figure S3. Shannon diversity index calculation of 16S rRNA gene sequencing data. Shannon diversity index was calculated using 16S rRNA bacterial community profiles for the three different 16S rRNA libraries tested in this study (27F-519R (region V1 + V2 + V3), 530F-926R (region V4 + V5) and 926F-1394R (region V6 + V7 + V8)). Sequencing data was analysed using the PE protocol. (PDF 16 kb)

Additional file 13: Figure S6. Primer alignment study of the most common bacterial taxa found in ELBW (P29F). **a** Representation of primers used in this study along the 16S bacterial rRNA gene. **b** Primer alignment study using 16S rRNA gene from *Bifidobacterium bifidum CP 010412* (isolated from Infloran) and the most common bacterial taxa found in an ELBW infant (P29F) with supplementation (*Staphylococcus epidermis* NR_074995, *Enterobacter cloacae* CP012165 and *Enterococcus faecalis* CP014949). We also included two strains of *Bifidobacterium* as control samples (*B.infantis* M58738.1 and *B.longum* ATCC 156697) All sequences are represented in 5′-3’orientation using UPAC nucleotide code, where Y = C or T, R = A or G, K = G or T, M = A or C (PDF 299 kb)

Additional file 14: Figure S7. PCR amplification using primers 530F-926R on 8 *Bifidobacterium* strains**.** PCR amplification targeting the bacterial 16S rRNA gene using primers 530F-926R on *Bifidobacterium* collection strains. DNA extracted from *B. bifidum* (isolated from the commercial probiotic supplementation) and seven different *Bifidobacterium* NCIMB collection strains, was amplified using primers 530F-926R. Positive controls for this study were a faecal metagenomic sample and a *L. acidophilus* strain (isolated from the probiotic supplementation). Pure water was used as negative control. Amplicon samples were run on 1% agarose gel for 30 min at 100 V. DNA was visualised under UV light after staining with ethidium bromide. All tested samples gave a PCR product. (PDF 351 kb)

Additional file 15: Figure S8. Melting curves of PCR amplicons from probiotic strains and bacterial preterm isolates. **a** Melting curves of PCR amplicons from probiotic strains *(Bifidobacterium bifidum* and *Lactobacillus acidophilus*) and bacterial preterm isolates (*Enterococcus faecium* and *Streptococcus infantarius*). Primers used to generate these amplicons were 530F-926R targeting region (V4 + V5). *Bifidobacterium bifidum* displayed the highest melting temperature. **b** Melting curve of PCR amplicon obtained from a mixed DNA sample (5 ng of *Bifidobacterium bifidum*, 5 ng of *Lactobacillus acidophilus*, 5 ng of *Enterococcus faecium* and 5 ng of *Streptococcus infantarius*). Primers used to generate these amplicons were 530F-926R targeting region (V4 + V5). Peak name (2) presents a melting temperature (Tm) similar to the melting temperature (Tm) obtained for *B. bifidum*. (PDF 100 kb)

Additional file 16: Figure S9. Comparison of bacterial profiles analysed by shotgun and 16S rRNA gene sequencing data using QIIME pipeline. Normalised data and relative abundance of the bacterial taxa was represented in percentages of number of reads. Bar colours represent different genus taxa, and bar lengths signify the relative abundance of each taxon. 16S rRNA bacterial profiles are named according to the different 16S rRNA hypervariable region amplified: (i) (V1 + V2 + V3, primers 27F-519R), (ii) (V4 + V5, primers 530F-926R) and (iii) (V6 + V7 + V8, primers 926F-1394R). a Bacterial community profiles determined by shotgun and 16S rRNA gene sequencing from an ELBW infant (sample AP8C) with no supplementation. b Bacterial community profiles determined by shotgun and 16S rRNA gene sequencing from an ELBW infant (sample P29F) with supplementation. c Bacterial community profiles determined by shotgun and 16S rRNA gene sequencing from a term baby (sample V3 J). More detailed information on the number of reads obtained by shotgun and 16S rRNA gene sequencing data can be found in Additional file 20. (PDF 36 kb)

Additional file 17: Figure S10. Shotgun functional profiles from two ELBW infants with /without supplementation and a term infant. Radial tree displaying shotgun functional profiles from an ELBW infant with no supplementation (sample AP8C, represented by the number 2 in the figure) an ELBW infant with supplementation (sample P29F, represented by the number 3 in the figure) and a term baby sample (sample V3 J, represented by the number 1 in the figure). Functional analysis was performed using the KEGG pathway analysis. (PDF 126 kb)

Additional file 18: Figure S11. PCoA plots based on 16S rRNA gene sequencing and shotgun data. **a** PCoA based on 16S rRNA gene sequencing data analysed using PE protocol. **b** PCoA based on 16S rRNA gene sequencing data analysed using QIIME. Blue circles represent sequencing data of preterm without supplementation (AP8C), yellow circles data of preterm with supplementation (P29F), and green circles data of term baby (V3 J). Each sample was analysed using three different 16S rRNA gene libraries (.27F, targets region (V1 + V2 + V3), .530F targets region (V4 + V5), and .926F targets region (V6 + V7 + V8)). Samples ended with (_shotgun) represents shotgun data used as ‘gold standard’ in this study. Samples from region (V4 + V5) presented the most distinct distribution when compared with shotgun data. More details of primers used for preparing these 16S rRNA libraries can be found in Methods section. (PDF 76 kb)

Additional file 19: Number of reads detected by PE and QIIME pipelines for the different hypervariable regions. (PDF 181 kb)

Additional file 20: Number of reads shotgun and 16S rRNA data (PE versus QIIME). (PDF 286 kb)

## Abbreviations

bp: base pair
ELBW: extremely low birth weight
NEC: necrotising enterocolitis
NGS: next generation sequencing
NICU: neonatal intensive care unit
OTU: operational taxonomic unit
PCR: polymerase chain reaction
PE: paired end protocol
V: hypervariable region of the 16S rRNA gene
