# Supplementary material for: Optimisation of 16S rRNA gut microbiota profiling of extremely low birth weight infants
Source: BMC Genomics. 2017 Nov 2;18:841. doi: 10.1186/s12864-017-4229-x (PMC5668952; doi:10.1186/s12864-017-4229-x)
Supplement: Supplementary file 1 — Subject details and metadata. (PDF 202 kb) [file 12864_2017_4229_MOESM1_ESM.pdf]

Table S1. Subject details and metadata.

Sample	Hospital	Birth mode	Term at week (weeks)	Birth weight (g)	Probiotic treatment (Infloran®)	Formula or breastfed	Sample collection[§] (Days after DOB)
AP1E	RH[†]	Vaginal	25	830	No	Breastfed	16
AP5D	RH	Vaginal	25	800	No	Breastfed	12
AP25E	RH	Vaginal	25	786	No	Breastfed	18
AP8C	RH	Vaginal	23	576	No	Breastfed	21
AP10B	RH	Vaginal	26	710	No	Breastfed	30
P31B	NNUH[‡]	Vaginal	23	605	Yes	Breastfed	16
P29F[*]	NNUH	Vaginal	26	1000	Yes	Breastfed	12
P30N[*]	NNUH	Vaginal	26	960	Yes	Breastfed	15
P35C	NNUH	Vaginal	23	565	Yes	Breastfed	16
P66F	NNUH	Vaginal	26	670	Yes	Breastfed	20
V3J	NNUH	Vaginal	40	3500	No	Breastfed	58
V2A	NNUH	Vaginal	40	3320	No	Breastfed	60
V3ZC	NNUH	Vaginal	40	3500	No	Breastfed[#]	365

[*]Baby P29F and P30N were twins.
[†]Rosie Hospital
[‡]Norfolk and Norwich University Hospital
[§]Faecal samples were collected in a stool container and stored at 4^0 C. DNA was extracted within 4 hours of collection.
[#] Exclusively breastfed baby until six month old.
DOB: date of birth
