# Supplementary material for: Optimisation of 16S rRNA gut microbiota profiling of extremely low birth weight infants
Source: BMC Genomics. 2017 Nov 2;18:841. doi: 10.1186/s12864-017-4229-x (PMC5668952; doi:10.1186/s12864-017-4229-x)
Supplement: Supplementary file 2 — Primers used in 16S rRNA sequencing library. (PDF 137 kb) [file 12864_2017_4229_MOESM2_ESM.pdf]

Table S2. Primers used in 16S rRNA sequencing library

Primer name	Shorthand	16S rRNA gene hypervariable region	Primer sequence
**27Fmod**	27F	V1+V2+V3	AGRGTTTGATCMTGGCTCAG
**ill519Rmod**	519R	V1+V2+V3	GTNTTACNGCGGCKGCTG
**530F**	530F	V4+V5	GTGCCAGCMGCNGCGG
**bac926R**	926R	V4+V5	CCGTCAATTYYTTTRAGTTT
**926F**	926F	V6+V7+V8	AAACTYAAAKGAATTGACGG
**bac1394R**	1394R	V6+V7+V8	ACGGGCGGTGTGTRC
