# Supplementary material for: Optimisation of 16S rRNA gut microbiota profiling of extremely low birth weight infants
Source: BMC Genomics. 2017 Nov 2;18:841. doi: 10.1186/s12864-017-4229-x (PMC5668952; doi:10.1186/s12864-017-4229-x)
Supplement: Supplementary file 3 — Scripts used to run the QIIME pipeline. (PDF 80 kb) [file 12864_2017_4229_MOESM3_ESM.pdf]

Commands:

1. Read assembly/merge using PEAR on SLURM

for j in AP25E_S5_L001 AP5D_S5_L001 AP8C_S5_L001 P29F_S5_L001 P30N_S5_L001 P31B_S5_L001 P35C_S5_L001 V2A_S5_L001 V3J_S5_L001;do sbatch --wrap " pear -f "$j"_R1_001.fastq -r "$j"_R2_001.fastq -q 40 -b 33 -o $j-merged.fq" -c 2 --mem=4GB -o out_merge -e error_merge -J PEAR;done

Verbose out: settings
Forward reads file.................: AP1E_S5_L001_R1_001.fastq
Reverse reads file.................: AP1E_S5_L001_R2_001.fastq
PHRED..............................: 33
Using empirical frequencies........: YES
Statistical method.................: OES
Maximum assembly length............: 999999
Minimum assembly length............: 50
p-value............................: 0.010000
Quality score threshold (trimming).: 40
Minimum read size after trimming...: 1
Maximal ratio of uncalled bases....: 1.000000
Minimum overlap....................: 10
Scoring method.....................: Scaled score

QIIME

quality filtering
sbatch --wrap "split_libraries_fastq.py -i AP1E-merged.fq.assembled.fastq --sample_ids AP1E -o ./ -q 19 --barcode_type 'not-barcoded'" -c 2 --mem=2GB -J qiime -o outsplit -e error_split -p nbi-short

Chimera detection
export PATH=$PATH:/tgac/software/testing/usearch61/6.1/x86_64/bin/
sbatch --wrap "identify_chimeric_seqs.py -i seqs.fna -m usearch61 -o usearch_checked_chimeras/ -r ../../../../../Chimera_db/uchime_chimera.fa " -c 8 --mem=20GB -p nbi-short -o out_chimera -e error_chimera

Delete chimeras
sbatch --wrap "filter_fasta.py -f seqs.fna -o seqs_chimeras_filtered.fna -s usearch_checked_chimeras/chimeras.txt -n" -c 2 --mem=2GB -J del -o out_chimera_remove -e error_chimera_remove

Pick OTUs

pick_open_reference_otus.py -o qiimeOutput_pickOpenRefOtus/ -i seqs_chimeras_filtered.fna -f -r ~/Databases/Qiime_SILVA_128/ SILVA_128_QIIME_release/rep_set/rep_set_16S_only/97/97_otus_16S.fasta -s 0.1

PE analysis

fastx filtering on Raw reads

```
for j in AP1E_S5_L001_R1 AP1E_S5_L001_R2 AP25E_S5_L001_R1
AP25E_S5_L001_R2 AP5D_S5_L001_R1 AP5D_S5_L001_R2 AP8C_S5_L001_R1
AP8C_S5_L001_R2 P29F_S5_L001_R1 P29F_S5_L001_R2 P30N_S5_L001_R1
P30N_S5_L001_R2 P31B_S5_L001_R1 P31B_S5_L001_R2 P35C_S5_L001_R1
P35C_S5_L001_R2 V2A_S5_L001_R1 V2A_S5_L001_R2 V3J_S5_L001_R1
V3J_S5_L001_R2;do echo $j;fastq_quality_filter -Q33 -q 30 -p 50 -i "$j"_001.fastq -o
"$j"_001_fil.fastq;done
```

fastq to fasta conversion

```
awk '{if(NR%4==1) {printf(">%s\n",substr($0,2));} else if(NR%4==2) print;}'
"$j"_R1_001_fil.fastq > "$j”_R1_001_fil.fasta;
```

Do BLAST against SILVA128

```
sbatch --wrap " blastn -query "$j" -db /usr/users/FR009/caims/
Databases/SILVA_128/SILVA_128_SSURef_tax_silva -out "$j".out -
num_threads 2 -num_alignments 30 -num_descriptions 30" -c 2 --
mem=5GB -J $j -o out -e error -p nbi-long;
```

Do BLASTN to MEGAN RMA

```
blast2rma -i file.blastn -r Reads.fa -pof -p  -f BlastText -bm BlastN -o 91_2.rma6 -g2t ~/
caims/MEGAN_mapping_files/nucl-gi2taxid-August2016.bin
```
