# Supplementary material for: Optimisation of 16S rRNA gut microbiota profiling of extremely low birth weight infants
Source: BMC Genomics. 2017 Nov 2;18:841. doi: 10.1186/s12864-017-4229-x (PMC5668952; doi:10.1186/s12864-017-4229-x)
Supplement: Supplementary file 4 — Number of raw reads counts for shotgun and 16S rRNA gene sequencing data (PDF 112 kb) [file 12864_2017_4229_MOESM4_ESM.pdf]

Additional file 4. Number of raw reads counts for shotgun and 16S rRNA gene sequencing data

Samples for Shotgun sequencing

Baby ID	Shotgun samples	Raw_read count	in MEGAN (Paired when possible)
V3J	S1_1.fastq	9,536,453	8,420,963
	S1_2.fastq	9,536,453	
AP8C	S2_1.fastq	12,382,963	10,589,359
	S2_2.fastq	12,382,963	
P29F	S3_1.fastq	10,896,924	9,622,411
	S3_2.fastq	10,896,924	

Samples for 16S rRNA sequencing using PE

Baby ID	Shotgun samples	Raw_read count	After Quality filter	in MEGAN (Paired when possible)
	V1+V2+V3			
AP1E	AP1E.27F_R1.fastq	154,509	146,552	
	AP1E.27F_R2.fastq	154,509	124,575	108,497
AP25E	AP25E.27F_R1.fastq	155,207	147,971	
	AP25E.27F_R2.fastq	155,207	122,997	270,759
AP5D	AP5D.27F_R1.fastq	101,963	97,419	
	AP5D.27F_R2.fastq	101,963	80,605	178,024
AP8C	AP8C.27F_R1.fastq	83,883	80,074	
	AP8C.27F_R2.fastq	83,883	66,301	146,371
P29F	P29F.27F_R1.fastq	100,264	95,214	
	P29F.27F_R2.fastq	100,264	75,983	171,169
P30N	P30N.27F_R1.fastq	113,827	108,139	
	P30N.27F_R2.fastq	113,827	88,764	196,897

Samples for 16S rRNA sequencing using QIIME

Baby ID	Shotgun samples	Raw_read count	Assembly	After Quality filter	in MEGAN (Paired when possible)
	V1+V2+V3				
AP1E	AP1E.27F_R1.fastq	154,509	149,927	74,768	
	AP1E.27F_R2.fastq	154,509			73,286
AP25E	AP25E.27F_R1.fastq	155,207	149,309	66,989	
	AP25E.27F_R2.fastq	155,207			66,233
AP5D	AP5D.27F_R1.fastq	101,963	96,606	31,555	
	AP5D.27F_R2.fastq	101,963			29,589
AP8C	AP8C.27F_R1.fastq	83,883	79,707	28,356	
	AP8C.27F_R2.fastq	83,883			25,808
P29F	P29F.27F_R1.fastq	100,264	95,641	37,141	
	P29F.27F_R2.fastq	100,264			35,173
P30N	P30N.27F_R1.fastq	113,827	106,924	32,515	
	P30N.27F_R2.fastq	113,827			31,450

P31B	P31B.27F_R1.fastq	104,542	100,022	
	P31B.27F_R2.fastq	104,542	83,931	183,910
P35C	P35C.27F_R1.fastq	131,266	124,227	
	P35C.27F_R2.fastq	131,266	101,482	225,702
V2A	V2A.27F_R1.fastq	104,410	99,819	
	V2A.27F_R2.fastq	104,410	80,254	180,052
V3J	V3J.27F_R1.fastq	94,344	90,100	
	V3J.27F_R2.fastq	94,344	72,869	162,932
	V4+V5			
AP1E	AP1E.530F_R1.fastq	1,079,921	1,054,856	
	AP1E.530F_R2.fastq	1,079,921	940,830	1,995,544
AP25E	AP25E.530F_R1.fastq	542,529	527,076	
	AP25E.530F_R2.fastq	542,529	444,981	967,069
AP5D	AP5D.530F_R1.fastq	754,988	737,055	
	AP5D.530F_R2.fastq	754,988	648,579	1,385,492
AP8C	AP8C.530F_R1.fastq	489,498	477,261	
	AP8C.530F_R2.fastq	489,498	415,314	892,360
P29F	P29F.530F_R1.fastq	469,124	457,067	
	P29F.530F_R2.fastq	469,124	394,279	846,302
P30N	P30N.530F_R1.fastq	576,331	564,022	
	P30N.530F_R2.fastq	576,331	493,048	1,056,787
P31B	P31B.530F_R1.fastq	423,862	413,347	
	P31B.530F_R2.fastq	423,862	363,728	776,976
P35C	P35C.530F_R1.fastq	492,809	483,177	
	P35C.530F_R2.fastq	492,809	429,850	912,978
V2A	V2A.530F_R1.fastq	679,918	662,228	
	V2A.530F_R2.fastq	679,918	574,822	1,236,324
V3J	V3J.530F_R1.fastq	418,955	408,725	
	V3J.530F_R2.fastq	418,955	356,165	763,712

P31B	P31B.27F_R1.fastq	104,542	100,864	44,990	
	P31B.27F_R2.fastq	104,542			41,677
P35C	P35C.27F_R1.fastq	131,266	122,234	36,039	
	P35C.27F_R2.fastq	131,266			34,051
V2A	V2A.27F_R1.fastq	104,410	102,296	59,276	
	V2A.27F_R2.fastq	104,410			57,969
V3J	V3J.27F_R1.fastq	94,344	92,210	51,365	
	V3J.27F_R2.fastq	94,344			46,407
	V4+V5				
AP1E	AP1E.530F_R1.fastq	1,079,921	1,078,121	1,052,924	
	AP1E.530F_R2.fastq	1,079,921			1,028,760
AP25E	AP25E.530F_R1.fastq	542,529	541,235	521,658	
	AP25E.530F_R2.fastq	542,529			504,693
AP5D	AP5D.530F_R1.fastq	754,988	753,758	733,507	
	AP5D.530F_R2.fastq	754,988			715,114
AP8C	AP8C.530F_R1.fastq	489,498	488,558	474,597	
	AP8C.530F_R2.fastq	489,498			450,256
P29F	P29F.530F_R1.fastq	469,124	467,292	452,341	
	P29F.530F_R2.fastq	469,124			430,128
P30N	P30N.530F_R1.fastq	576,331	575,366	560,498	
	P30N.530F_R2.fastq	576,331			546,603
P31B	P31B.530F_R1.fastq	423,862	423,043	412,194	
	P31B.530F_R2.fastq	423,862			396,517
P35C	P35C.530F_R1.fastq	492,809	491,243	479,886	
	P35C.530F_R2.fastq	492,809			462,862
V2A	V2A.530F_R1.fastq	679,918	678,285	658,393	
	V2A.530F_R2.fastq	679,918			635,528
V3J	V3J.530F_R1.fastq	418,955	417,757	405,838	
	V3J.530F_R2.fastq	418,955			384,789

	V6+V7+V8			
AP1E	AP1E.926F_R1.fastq	148,260	142,900	
	AP1E.926F_R2.fastq	148,260	117,216	260,092
AP25E	AP25E.926F_R1.fastq	139,882	135,615	
	AP25E.926F_R2.fastq	139,882	115,599	251,211
AP5D	AP5D.926F_R1.fastq	98,244	94,729	
	AP5D.926F_R2.fastq	98,244	80,025	174,754
AP8C	AP8C.926F_R1.fastq	99,080	95,770	
	AP8C.926F_R2.fastq	99,080	81,301	177,054
P29F	P29F.926F_R1.fastq	66,289	63,985	
	P29F.926F_R2.fastq	66,289	50,986	114,970
P30N	P30N.926F_R1.fastq	101,995	98,807	
	P30N.926F_R2.fastq	101,995	84,480	183,269
P31B	P31B.926F_R1.fastq	131,601	126,617	
	P31B.926F_R2.fastq	131,601	100,729	227,339
P35C	P35C.926F_R1.fastq	120,933	116,860	
	P35C.926F_R2.fastq	120,933	96,829	213,685
V2A	V2A.926F_R1.fastq	99,339	94,646	
	V2A.926F_R2.fastq	99,339	68,738	163,384
V3J	V3J.926F_R1.fastq	126,399	121,345	
	V3J.926F_R2.fastq	126,399	92,676	213,519

	V6+V7+V8				
AP1E	AP1E.926F_R1.fastq	148,260	146,736	99,832	
	AP1E.926F_R2.fastq	148,260			94,851
AP25E	AP25E.926F_R1.fastq	139,882	138,988	103,182	
	AP25E.926F_R2.fastq	139,882			101,554
AP5D	AP5D.926F_R1.fastq	98,244	97,503	69,336	
	AP5D.926F_R2.fastq	98,244			62,979
AP8C	AP8C.926F_R1.fastq	99,080	98,425	71,562	
	AP8C.926F_R2.fastq	99,080			59,316
P29F	P29F.926F_R1.fastq	66,289	65,658	41,768	
	P29F.926F_R2.fastq	66,289			36,853
P30N	P30N.926F_R1.fastq	101,995	101,345	74,108	
	P30N.926F_R2.fastq	101,995			69,663
P31B	P31B.926F_R1.fastq	131,601	129,856	76,795	
	P31B.926F_R2.fastq	131,601			31,552
P35C	P35C.926F_R1.fastq	120,933	119,842	82,133	
	P35C.926F_R2.fastq	120,933			61,947
V2A	V2A.926F_R1.fastq	99,339	97,790	48,016	
	V2A.926F_R2.fastq	99,339			45,181
V3J	V3J.926F_R1.fastq	126,399	124,563	70,010	
	V3J.926F_R2.fastq	126,399			49,171
