# Supplementary material for: Optimisation of 16S rRNA gut microbiota profiling of extremely low birth weight infants
Source: BMC Genomics. 2017 Nov 2;18:841. doi: 10.1186/s12864-017-4229-x (PMC5668952; doi:10.1186/s12864-017-4229-x)
Supplement: Supplementary file 5 — Bifidobacterium strains used for validating primers 530F-926R using PCR (PDF 198 kb) [file 12864_2017_4229_MOESM5_ESM.pdf]

Table S3. *Bifidobacterium* strains used for validating primers 530F-926R using PCR

***Bifidobacterium* strains**	**NCIMB collection number**	**Isolated from**	**Reference**
B. longum	8809	Nursing stools	(1)
B.bifidum	13922	Not described	(2)
B.catenatum	702239	Human faeces	(3)
B.angulatum	702236	Human faeces	(3)
B.adolescentis	702204	Adult intestine	(4)
B.breve	8807	Infant intestine	(4)
B.infantis	702255	Infant intestine	(4)

References:

1. Mattarelli P, Bonaparte C, Pot B, Biavati B. Proposal to reclassify the three biotypes of Bifidobacterium longum as three subspecies: Bifidobacterium longum subsp. longum subsp. nov., Bifidobacterium longum subsp. infantis comb. nov. and Bifidobacterium longum subsp. suis comb. nov. International journal of systematic and evolutionary microbiology. 2008;58(Pt 4):767-72.
2. 1924 TO-J. 1924. http://bacdive.dsmz.de/resultpdf.php?resultid=1691/.
3. Scardovi V, Crociani F. Bifidobacterium catenulatum, Bifidobacterium dentium, and Bifidobacterium angulatum: three new species and their deoxyribonucleic acid homology relationships. International journal of systematic and evolutionary microbiology. 1974;24(1):6-20.
4. Reuter. 1963. http://bacdive.dsmz.de/resultpdf.php?resultid=1683/.
