# Supplementary material for: Optimisation of 16S rRNA gut microbiota profiling of extremely low birth weight infants
Source: BMC Genomics. 2017 Nov 2;18:841. doi: 10.1186/s12864-017-4229-x (PMC5668952; doi:10.1186/s12864-017-4229-x)
Supplement: Supplementary file 6 — DNA yield from different DNA extraction methods. (PDF 306 kb) [file 12864_2017_4229_MOESM6_ESM.pdf]

Table S4. DNA yield from different DNA extraction methods.

Sample	Extraction method	Qubit (ng/μl)
ELBW infant no probiotics (AP10B)	FastDNA™ SPIN Kit for Soil (3 min. bead-beating)	2.25
	FastDNA™ SPIN Kit for Soil (30 s bead-beating)	1.97
	QIAamp DNA stool mini kit	<0.0005
	Enzymatic lysis and QIAamp DNA stool mini kit	0.0146
ELBW infant with probiotics (P66F)	FastDNA™ SPIN Kit for Soil (3 min. bead-beating)	13.8
	FastDNA™ SPIN Kit for Soil (30 s bead-beating)	7.38
	QIAamp DNA stool mini kit	<0.0005
	Enzymatic lysis and QIAamp DNA stool mini kit	0.0156
Term baby (V3ZC)	FastDNA™ SPIN Kit for Soil (3 min. bead-beating)	21.4
	FastDNA™ SPIN Kit for Soil (30 s bead-beating)	7.7
	QIAamp DNA stool mini kit	0.0164
	Enzymatic lysis and QIAamp DNA stool mini kit	0.77
