# Supplementary figures and images for: Optimisation of 16S rRNA gut microbiota profiling of extremely low birth weight infants
Source: BMC Genomics. 2017 Nov 2;18:841. doi: 10.1186/s12864-017-4229-x (PMC5668952; doi:10.1186/s12864-017-4229-x)

a

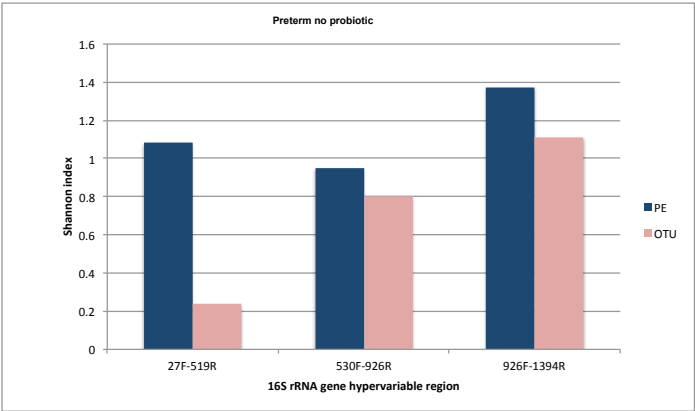

b

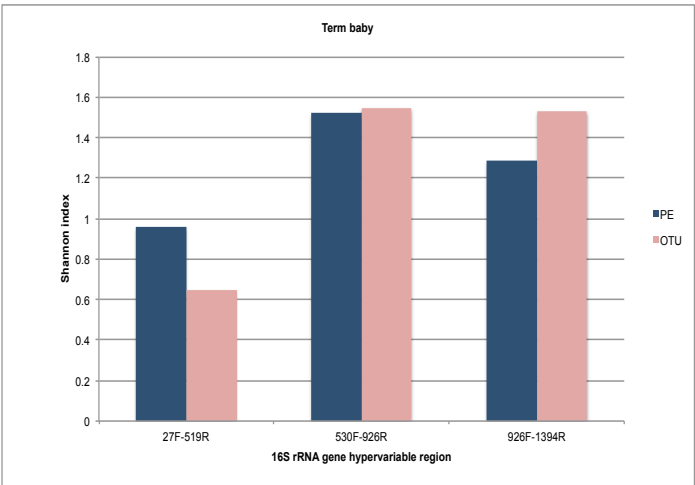

Supplement: Supplementary file 7 — Shannon diversity index on 16S rRNA gene sequencing data analysed using OTU and PE protocol. Shannon diversity index was calculated using 16S rRNA bacterial community profiles for sample AP1E (ELBW infant without probiotic supplementation) and sample V3 J (term infant). a Shannon diversity indexes comparison of three different 16S rRNA libraries (27F-519R (region V1 + V2 + V3), 530F-926R (region V4 + V5) and 926F-1394R (region V6 + V7 + V8)) using OTU and PE protocol pipelines for sample AP1E (preterm no supplementation). b Shannon diversity indexes comparison of three different 16S rRNA libraries (27F-519R (region V1 + V2 + V3), 530F-926R (region V4 + V5) and 926F-1394R (region V6 + V7 + V8)) using OTU and PE protocol pipelines for sample V3 J (term infant). (PDF 74 kb) [file 12864_2017_4229_MOESM7_ESM.pdf]

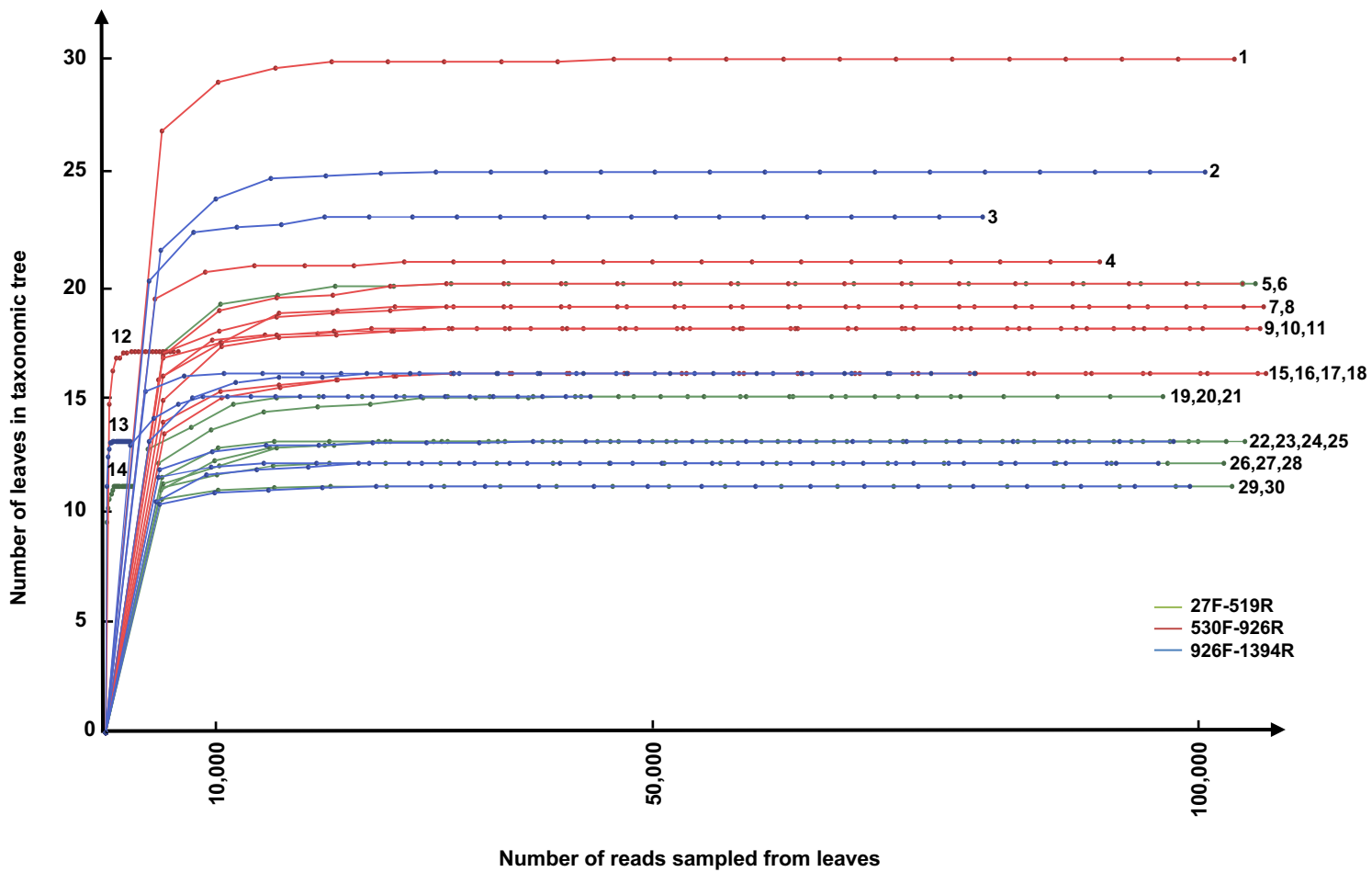

Supplement: Supplementary file 9 — Rarefaction curves 16S rRNA gene sequencing data. Rarefaction curves representing number of species (leaves) detected at genus level versus number of reads sampled. Three different 16S rRNA gene sequencing data were used for this study: i) green curves represent sequencing data from 16S rRNA library prepared using primers 27F-519R, ii) red curves represent sequencing data from 16S rRNA library prepared using primers 530F-926R and iii) blue curves represent sequencing data from 16S rRNA library prepared using primers 926F-1394R. Rarefaction curves are labelled with numbers to differentiate among the samples used in the study: 1 (V2A.530F), 2 (V2A.926F), 3 (V3 J.926F), 4 (AP8C.530F), 5 (V2AJ.27F), 6 (AP5D.530F), 7 (P35C.530F), 8 (V3 J.530F), 9 (P29F.530F), 10 (P31B.530F), 11 (P30N.530F), 12 (AP25E.530F), 13 (AP25E.926F), 14 (AP25E. 27F), 15 (AP8C.926F), 16 (P35C.926F), 17 (V3 J.27F), 18 (P31B.926F), 19 (AP1E.530F), 20 (P31B.27F), 21(P29F.27F), 22 (P30N.27F), 23 (AP5D.926F), 24 (AP5D.27F), 25 (P29F.926F), 26 (AP1E.926F),27 (AP1E.27F), 28 (AP8C.27F), 29 (P30N.926F), 30 (P35C.27F). Numbers 12, 13 and 14 correspond to sample AP25E where majority of sequenced reads assigned at family level (i.e. Enterobacteriaceae). (PDF 62 kb) [file 12864_2017_4229_MOESM9_ESM.pdf]

PC1 (65.5%) vs PC2 (23.9%)

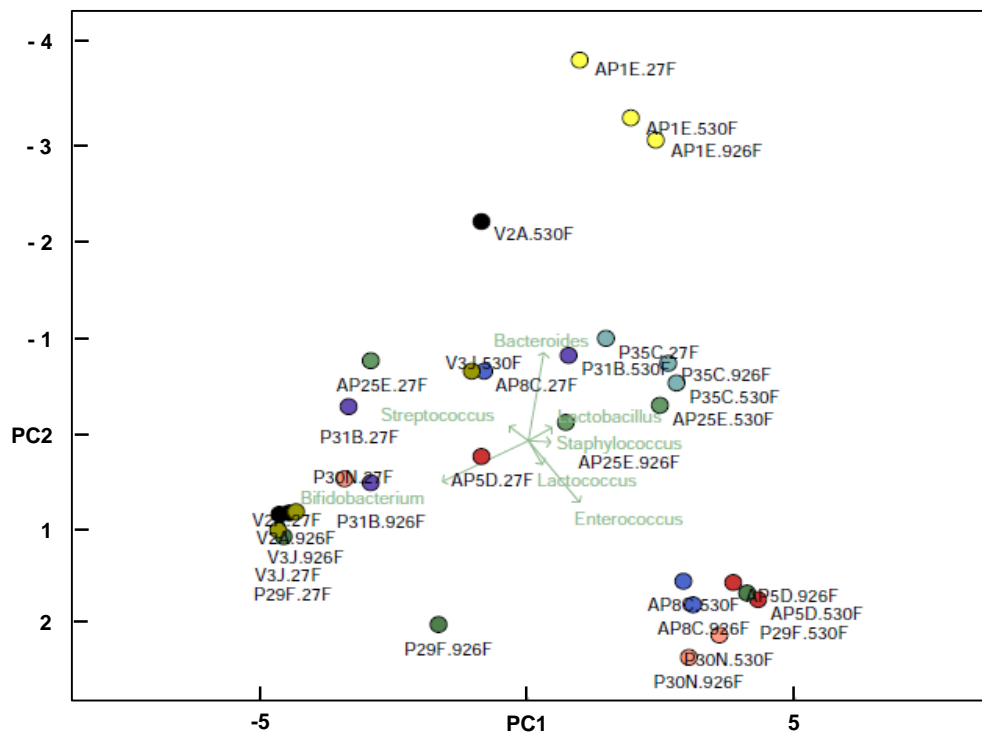

Supplement: Supplementary file 11 — Principal Coordinate Analysis (PCoA) based on 16S rRNA community profiles analysed using QIIME of the hypervariable regions tested. PCoA was performed based on the taxonomic assignments obtained from the 16S rRNA gene sequencing libraries analysed. Samples used for this plot were classified in main three groups: (i) preterms without supplementation (AP1E, AP5D, AP8C, AP25C), (ii) preterms with supplementation (P29F, P30N, P31B, P35C), and (iii) term baby samples (V2A, V3 J). Samples names are coded highlighting the 16S rRNA gene library they belong. Sample names ending in (.27F) belong to 16S rRNA gene library prepared using using primers 27F-519R (target region V1 + V2 + V3), sample names ending in (.530F) belong to 16S rRNA gene library prepared using primers 530F-926R (region V4 + V5), and sample names ending in (.926F) belong to 16S rRNA gene library amplified using primers 926F-1394R (region V6 + V7 + V8). PCoA plot indicates that distribution of samples targeting (V4 + V5) region was distinct from samples targeting (V1 + V2 + V3) and (V6 + V7 + V8). (PDF 37 kb) [file 12864_2017_4229_MOESM11_ESM.pdf]

Shannon diversity index

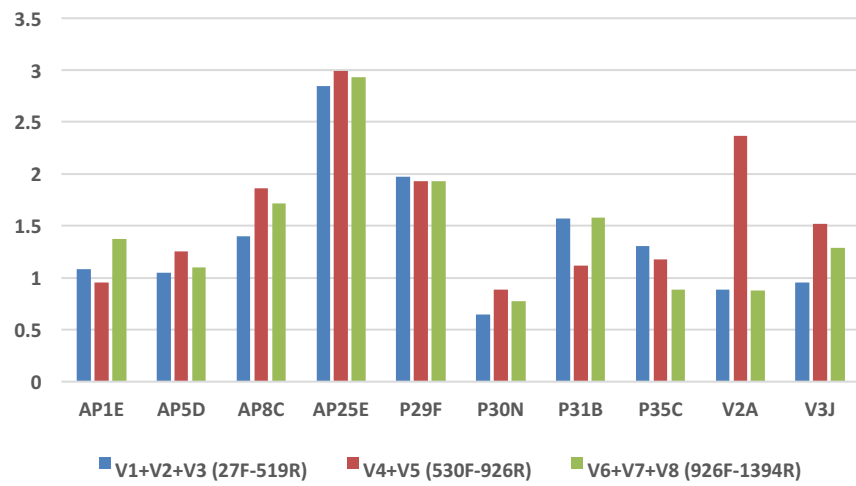

Supplement: Supplementary file 12 — Shannon diversity index calculation of 16S rRNA gene sequencing data. Shannon diversity index was calculated using 16S rRNA bacterial community profiles for the three different 16S rRNA libraries tested in this study (27F-519R (region V1 + V2 + V3), 530F-926R (region V4 + V5) and 926F-1394R (region V6 + V7 + V8)). Sequencing data was analysed using the PE protocol. (PDF 16 kb) [file 12864_2017_4229_MOESM12_ESM.pdf]

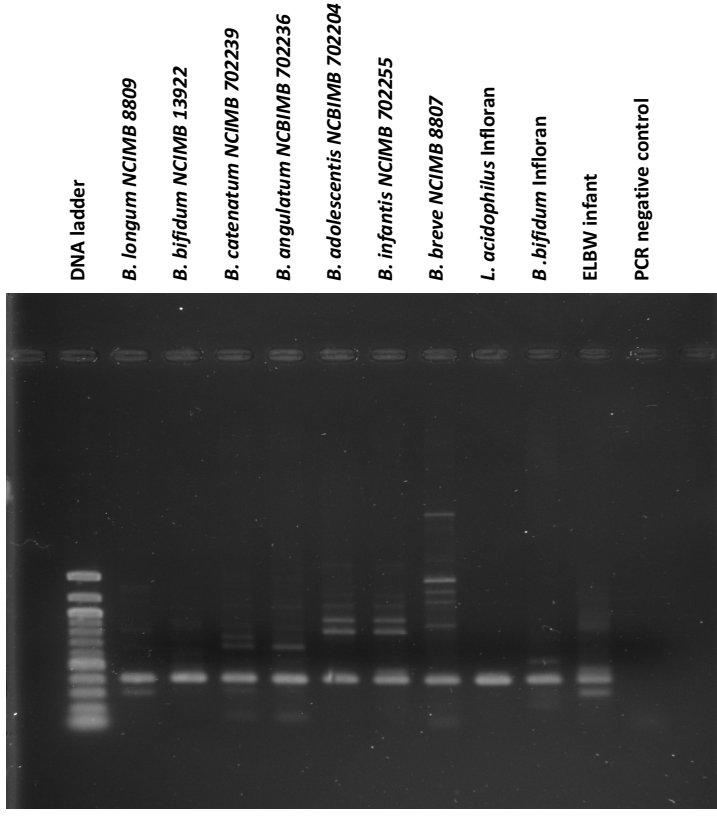

Supplement: Supplementary file 14 — PCR amplification using primers 530F-926R on 8 Bifidobacterium strains. PCR amplification targeting the bacterial 16S rRNA gene using primers 530F-926R on Bifidobacterium collection strains. DNA extracted from B. bifidum (isolated from the commercial probiotic supplementation) and seven different Bifidobacterium NCIMB collection strains, was amplified using primers 530F-926R. Positive controls for this study were a faecal metagenomic sample and a L. acidophilus strain (isolated from the probiotic supplementation). Pure water was used as negative control. Amplicon samples were run on 1% agarose gel for 30 min at 100 V. DNA was visualised under UV light after staining with ethidium bromide. All tested samples gave a PCR product. (PDF 351 kb) [file 12864_2017_4229_MOESM14_ESM.pdf]

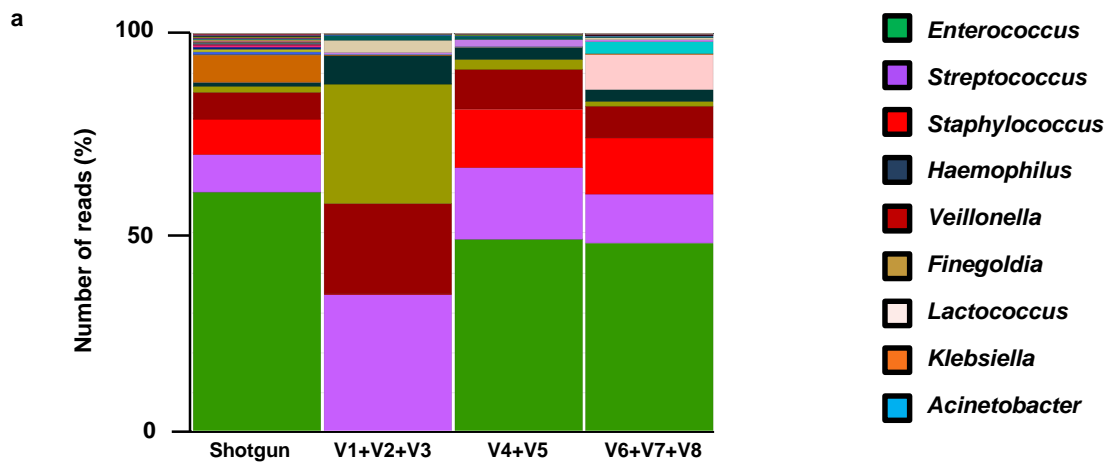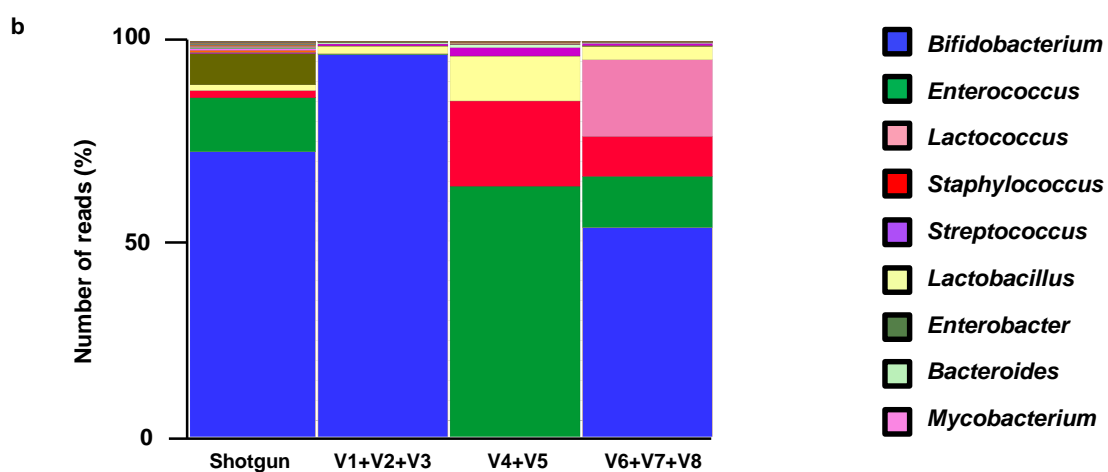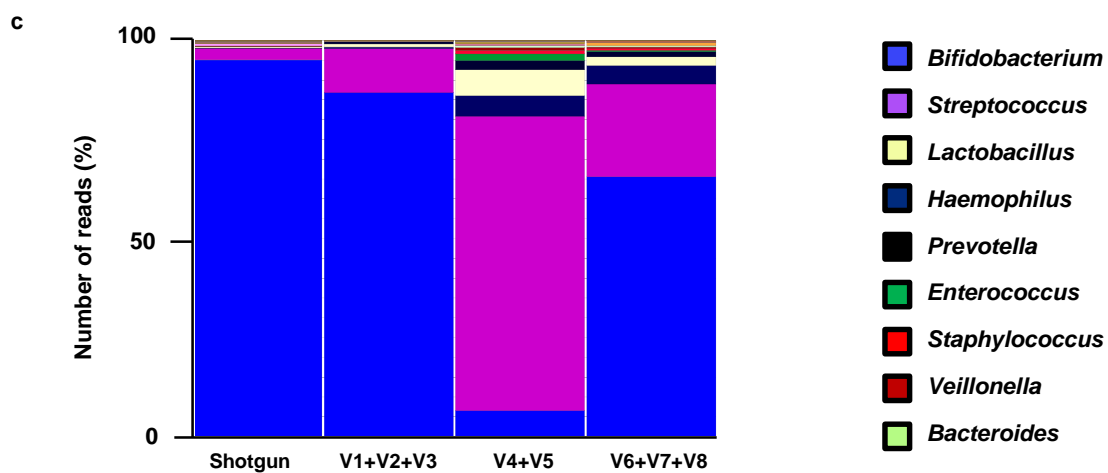

Supplement: Supplementary file 16 — Comparison of bacterial profiles analysed by shotgun and 16S rRNA gene sequencing data using QIIME pipeline. Normalised data and relative abundance of the bacterial taxa was represented in percentages of number of reads. Bar colours represent different genus taxa, and bar lengths signify the relative abundance of each taxon. 16S rRNA bacterial profiles are named according to the different 16S rRNA hypervariable region amplified: (i) (V1 + V2 + V3, primers 27F-519R), (ii) (V4 + V5, primers 530F-926R) and (iii) (V6 + V7 + V8, primers 926F-1394R). a Bacterial community profiles determined by shotgun and 16S rRNA gene sequencing from an ELBW infant (sample AP8C) with no supplementation. b Bacterial community profiles determined by shotgun and 16S rRNA gene sequencing from an ELBW infant (sample P29F) with supplementation. c Bacterial community profiles determined by shotgun and 16S rRNA gene sequencing from a term baby (sample V3 J). More detailed information on the number of reads obtained by shotgun and 16S rRNA gene sequencing data can be found in Additional file 20. (PDF 36 kb) [file 12864_2017_4229_MOESM16_ESM.pdf]

KEGG profileComparison Radial tree plot

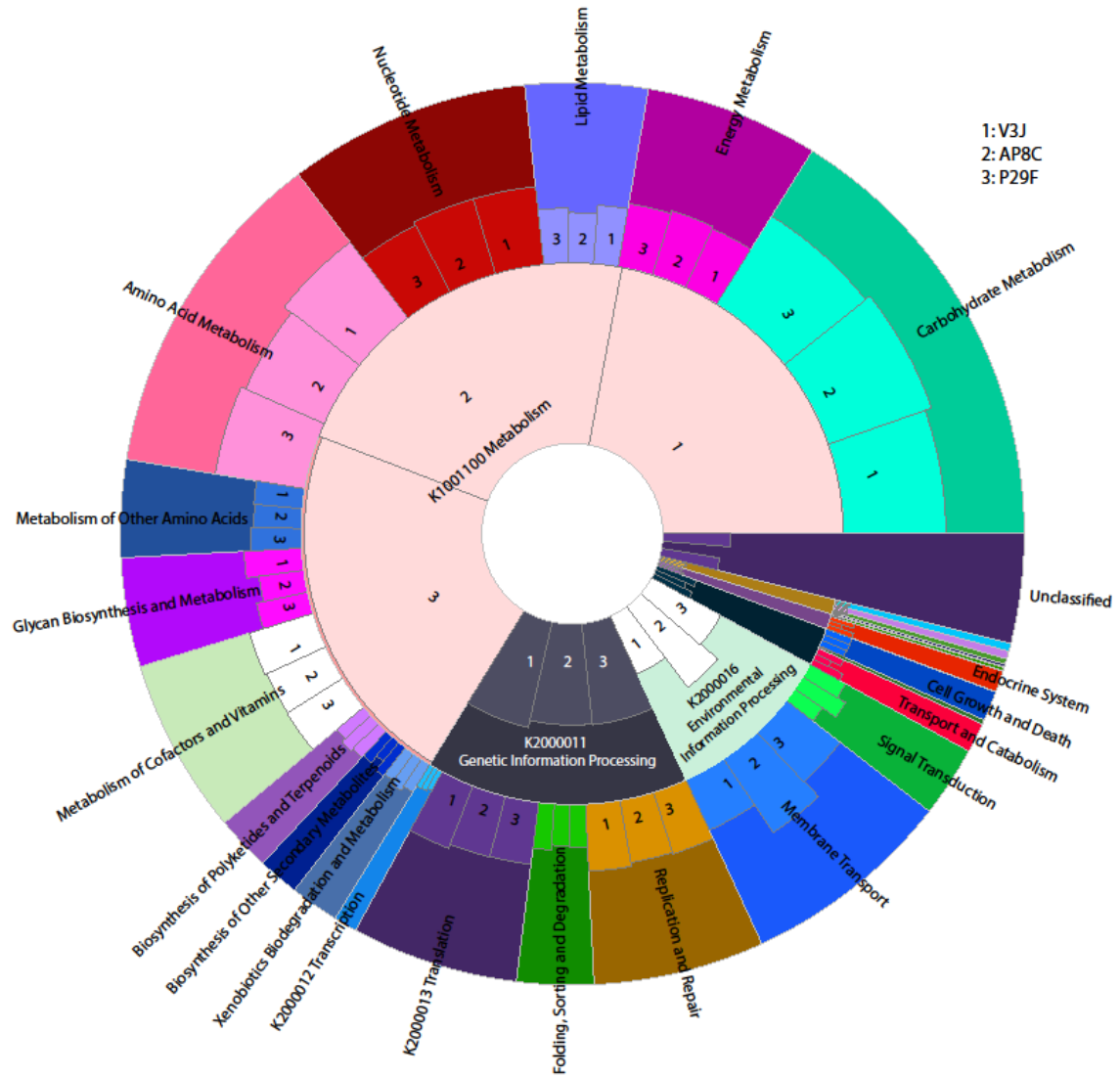

Supplement: Supplementary file 17 — Shotgun functional profiles from two ELBW infants with /without supplementation and a term infant. Radial tree displaying shotgun functional profiles from an ELBW infant with no supplementation (sample AP8C, represented by the number 2 in the figure) an ELBW infant with supplementation (sample P29F, represented by the number 3 in the figure) and a term baby sample (sample V3 J, represented by the number 1 in the figure). Functional analysis was performed using the KEGG pathway analysis. (PDF 126 kb) [file 12864_2017_4229_MOESM17_ESM.pdf]

a

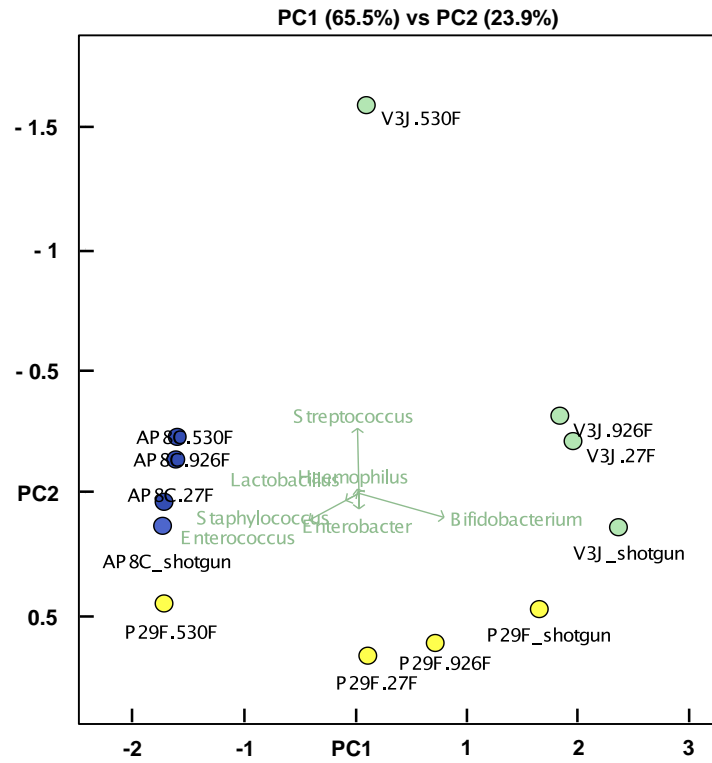

b

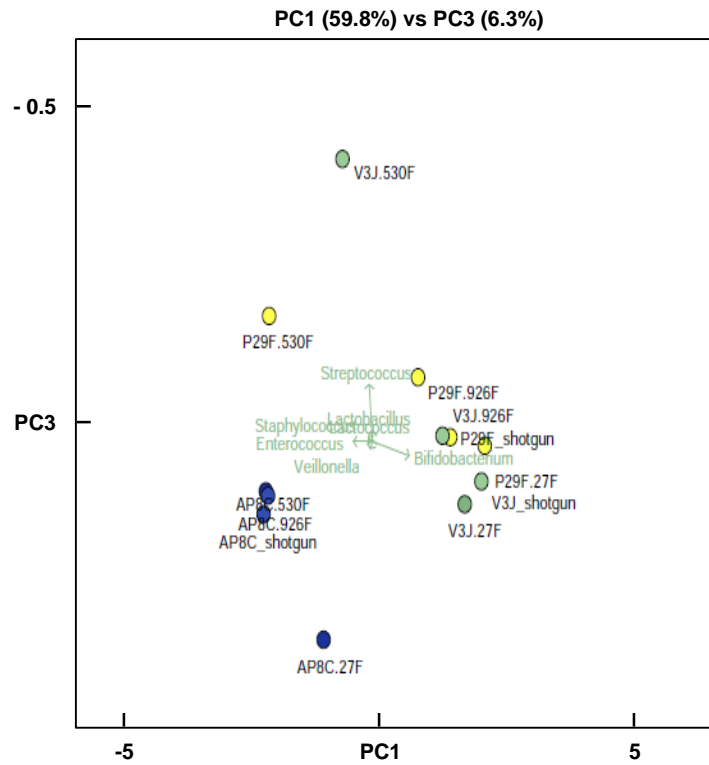

Supplement: Supplementary file 18 — PCoA plots based on 16S rRNA gene sequencing and shotgun data. a PCoA based on 16S rRNA gene sequencing data analysed using PE protocol. b PCoA based on 16S rRNA gene sequencing data analysed using QIIME. Blue circles represent sequencing data of preterm without supplementation (AP8C), yellow circles data of preterm with supplementation (P29F), and green circles data of term baby (V3 J). Each sample was analysed using three different 16S rRNA gene libraries (.27F, targets region (V1 + V2 + V3), .530F targets region (V4 + V5), and .926F targets region (V6 + V7 + V8)). Samples ended with (_shotgun) represents shotgun data used as ‘gold standard’ in this study. Samples from region (V4 + V5) presented the most distinct distribution when compared with shotgun data. More details of primers used for preparing these 16S rRNA libraries can be found in Methods section. (PDF 76 kb) [file 12864_2017_4229_MOESM18_ESM.pdf]
