# Supplementary material for: Optimisation of 16S rRNA gut microbiota profiling of extremely low birth weight infants
Source: BMC Genomics. 2017 Nov 2;18:841. doi: 10.1186/s12864-017-4229-x (PMC5668952; doi:10.1186/s12864-017-4229-x)
Supplement: Supplementary file 8 — Number of reads obtained by PE and QIIME for samples V3 J and AP1E. (PDF 76 kb) [file 12864_2017_4229_MOESM8_ESM.pdf]

Additional file 8 - Number of reads obtained by PE and QIIME for samples V3J and AP1E

PE protocol V3J	V3J.27F	V3J.530F	V3J.926F
#Datasets			
Bifidobacterium	128700	10043	90671
Streptococcus	23239	115525	18838
Lactobacillus	3010	10284	2407
Prevotella	1040	3668	1302
Enterococcus	1030	2847	1126
Haemophilus	602	8166	4105
Staphylococcus	595	1320	580
Bacteroides	337	883	365
Enterobacter	96	208	150
Citrobacter	69	2120	0
Escherichia	66	97	137
Acinetobacter	34	20	0
Lachnoclostridium	30	55	25
Corynebacterium	26	0	56
Dolosigranulum	17	0	60
Salmonella	0	0	27
Shigella	0	19	171
Actinomyces	0	149	31
Gardnerella	0	0	46
Rothia	0	0	208
Propionibacterium	0	105	0
Gemella	0	39	0
Granulicatella	0	17	0
Candida	0	0	97

QIIME protocol V3J	V3J.27F	V3J.530F	V3J.926F
#Datasets			
Bifidobacterium	39844	2976	29285
Streptococcus	5082	33485	10433
Lactobacillus	364	2924	976
Prevotella	277	1061	589
Haemophilus	155	2350	2106
Bacteroides	95	251	166
Finegoldia	13	153	24
Anaerococcus	12	126	63
Veillonella	9	386	130
Bacillus	8	0	0
Acinetobacter	6	5	5
Corynebacterium	5	72	20
Rothia	2	0	81
Peptoniphilus	2	85	22
Trabulsiella	0	0	3
Erwinia	0	1	0
Pseudomonas	0	1	0
Actinomyces	0	42	11
Propionibacterium	0	30	4
Atopobium	0	1	0
Gemella	0	4	3
Staphylococcus	0	346	212
Alloiococcus	0	0	28
Granulicatella	0	22	0
Enterococcus	0	767	145
Lactococcus	0	0	340
Clostridium	0	4	0
Peptostreptococcus	0	1	0
Dialister	0	6	5

PE protocol AP1E	AP1E.27F	AP1E.530F	AP1E.926F
#Datasets			
Bacteroides	74173	87074	64580
Staphylococcus	17209	13872	25325
Lachnoclostridium	3645	3012	3572
Corynebacterium	696	12	2534
Enterococcus	517	1021	546
Bifidobacterium	353	47	398
Lactobacillus	182	430	235
Streptococcus	139	583	131
Erysipelatoclostridium	56	331	634
Acinetobacter	29	0	0
Enterobacter	21	28	28
Prevotella	14	23	0
Actinomyces	0	41	0
Citrobacter	0	14	0
Haemophilus	0	13	13
Lachnoanaerobaculum	0	19	0
Salinicoccus	0	0	25

QIIME protocol AP1E	AP1E.27F	AP1E.530F	AP1E.926F
#Datasets			
Bacteroides	53891	59245	48541
Corynebacterium	1364	615	1784
Bifidobacterium	214	31	200
Staphylococcus	55	8817	17975
Lactobacillus	35	265	108
Streptococcus	34	370	82
Paenibacillus	28	0	0
Acinetobacter	17	3	0
Prevotella	4	15	0
Finegoldia	4	8	0
Veillonella	2	31	12
Citrobacter	0	0	5
Haemophilus	0	8	11
Actinomyces	0	27	0
Propionibacterium	0	2	0
Salinicoccus	0	1	0
Granulicatella	0	125	0
Enterococcus	0	505	141
Lactococcus	0	0	330
Clostridium	0	3	0
Moryella	0	1	0
Peptostreptococcus	0	1	0
Anaerococcus	0	1	0
Peptoniphilus	0	1	0
Moryella	0	1	0
Peptostreptococcus	0	1	0
Anaerococcus	0	1	0
Peptoniphilus	0	1	0
