# Supplementary material for: Optimisation of 16S rRNA gut microbiota profiling of extremely low birth weight infants
Source: BMC Genomics. 2017 Nov 2;18:841. doi: 10.1186/s12864-017-4229-x (PMC5668952; doi:10.1186/s12864-017-4229-x)
Supplement: Supplementary file 10 — Comparison of taxonomic assignments among the 16S rRNA gene hypervariable regions tested using QIIME approach. Heat map displaying number of reads assigned to the most common bacterial taxa found in the study samples using QIIME bioinformatics pipeline. Top panel row divides the figure in the different regions of the 16 s rRNA gene analysed, namely: V1 + V2 + V3 (primers 27F-519R), V4 + V5 (primers 530F-926R) and V6 + V7 + V8 (primers 926F-1394R). The vertical axis of the panel indicates a selection of the 13 most common bacterial taxa found. The horizontal axis labels the different samples used in the study: preterms without supplementation (AP1E, AP5D, AP8C, AP25C), preterms with supplementation (P29F, P30N, P31B, P35C), and term baby samples (V2A, V3 J). The intensity of the green colour highlights the abundance of the number of reads found. Probiotic supplementation has been abbreviated to supplem. in the figure. Further information on the number of reads obtained for this study can be found in Additional file 19. (PDF 262 kb) [file 12864_2017_4229_MOESM10_ESM.pdf]

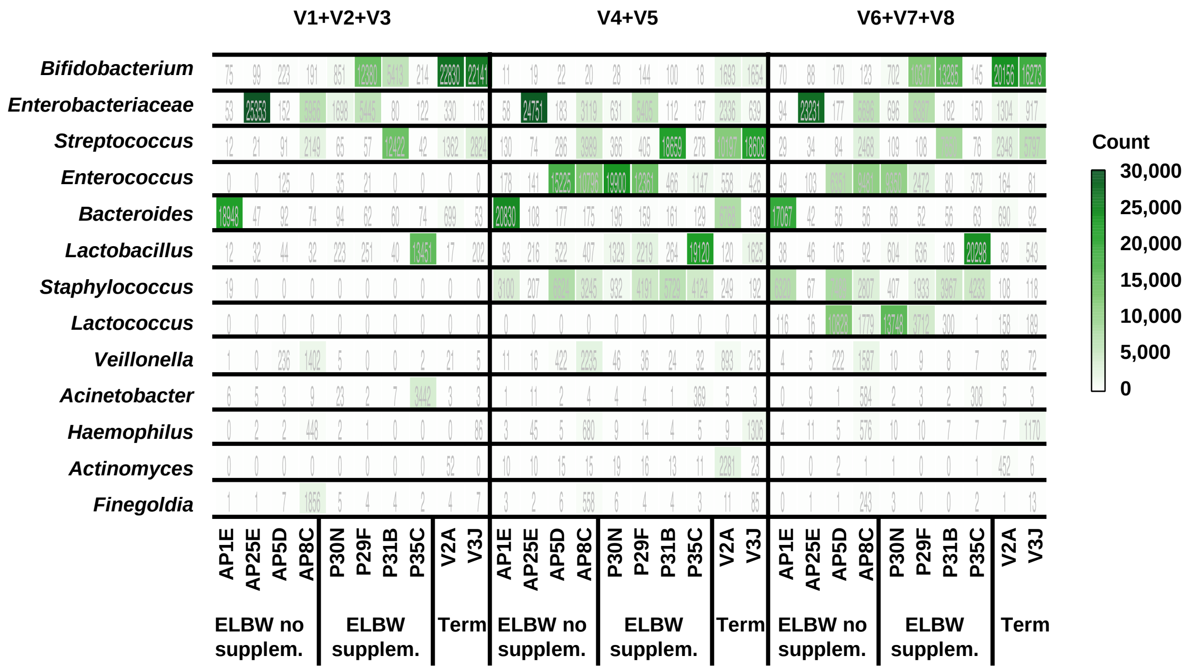
V1+V2+V3
V4+V5
V6+V7+V8
Bifidobacterium
Enterobacteriaceae
Streptococcus
Enterococcus
Bacteroides
Lactobacillus
Staphylococcus
Lactococcus
Veillonella
Acinetobacter
Haemophilus
Actinomyces
Finegoldia
AP1E
AP25E
AP5D
AP8C
P30N
P29F
P31B
P35C
V2A
V3J
ELBW no supplem.
ELBW supplem.
Term
Count
30,000
25,000
20,000
15,000
10,000
5,000
0
