# Supplementary material for: Optimisation of 16S rRNA gut microbiota profiling of extremely low birth weight infants
Source: BMC Genomics. 2017 Nov 2;18:841. doi: 10.1186/s12864-017-4229-x (PMC5668952; doi:10.1186/s12864-017-4229-x)
Supplement: Supplementary file 13 — Primer alignment study of the most common bacterial taxa found in ELBW (P29F). a Representation of primers used in this study along the 16S bacterial rRNA gene. b Primer alignment study using 16S rRNA gene from Bifidobacterium bifidum CP 010412 (isolated from Infloran) and the most common bacterial taxa found in an ELBW infant (P29F) with supplementation (Staphylococcus epidermis NR_074995, Enterobacter cloacae CP012165 and Enterococcus faecalis CP014949). We also included two strains of Bifidobacterium as control samples (B.infantis M58738.1 and B.longum ATCC 156697) All sequences are represented in 5′-3’orientation using UPAC nucleotide code, where Y = C or T, R = A or G, K = G or T, M = A or C (PDF 299 kb) [file 12864_2017_4229_MOESM13_ESM.pdf]

a

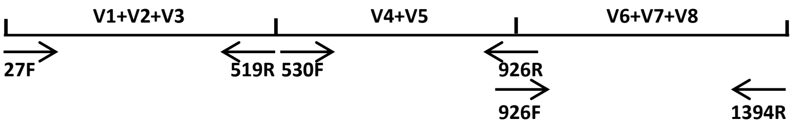

b

	22 … 41
16S rRNA *B. bifidum* CP010412	AGGGTTCGATTCTGGCTCAG
16S rRNA *S. epidermis* NR_074995	AGAGTTTGATCCTGGCTCAG
16S rRNA *E. cloacae* CP012165	AGAGTTTGATCATGGCTCAG
16S rRNA *E. faecalis* CP014949	AGAGTTTGATCCTGGCTCAG
16S rRNA *B. infantis* M58738.1	AGAGTTTGATCCTGGCTCAG
16S rRNA *B. longum* ATCC 156697	AGGGTTCGATTCTGGCTCAG
27Fmod primer	AGRGTTTGATCMTGGCTCAG
	* *

	536 … 553
16S rRNA *B. bifidum* CP010412	CAGCAGCCGCGGTAATAC
16S rRNA *S. epidermis* NR_074995	CAGCAGCCGCGGTAATAC
16S rRNA *E. cloacae* CP012165	CAGCAGCCGCGGTAATAC
16S rRNA *E. faecalis* CP014949	CAGCAGCCGCGGTAATAC
16S rRNA *B. infantis* M58738.1	CAGCNGCCGCGGTAATAC
16S rRNA *B. longum* ATCC 156697	CAGCAGCCGCGGTAATAC
519R primer	CAGCMGCCGCNGTAANAC

	540 … 555
16S rRNA *B. bifidum* CP010412	GTGCCAGCAGCCGCGG
16S rRNA *S. Epidermis* NR_074995	GTGCCAGCAGCCGCGG
16S rRNA *E. cloacae* CP012165	GTGCCAGCAGCAGCGG
16S rRNA *E. faecalis* CP014949	GTGCCAGCAGCCGCGG
16S rRNA *B. infantis* M58738.1	GTGCCAGCNGCCGCGG
16S rRNA *B. longum* ATCC 156697	GTGCCAGCAGCCGCGG
530F primer	GTGCCAGCMGCNGCGG

	930 … 949
16S rRNA *B. bifidum* CP010412	AAACTCAAAGAAATTGACGG
16S rRNA *S. Epidermis* NR_074995	AAACTCAAAGGAATTGACGG
16S rRNA *E. cloacae* CP012165	AAACTCAAATGAATTGACGG
16S rRNA *E. faecalis* CP014949	AAACTCAAAGGAATTGACGG
16S rRNA *B. infantis* M58738.1	AAACTCAAAGAAATTGACGG
16S rRNA *B. longum* ATCC 156697	AAACTCAAAGAAATTGACGG
bac926R primer	AAACTYAAARRAATTGACGG
	*

	930 … 949
16S rRNA *B. bifidum* CP010412	AAACTCAAAGAAATTGACG
16S rRNA *S. Epidermis* NR_074995	AAACTCAAAGGAATTGACG
16S rRNA *E. cloacae* CP012165	AAACTCAAATGAATTGACG
16S rRNA *E. faecalis* CP014949	AAACTCAAAGGAATTGACG
16S rRNA *B. infantis* M58738.1	AAACTCAAAGAAATTGACG
16S rRNA *B. longum* ATCC 156697	AAACTCAAAGAAATTGACG
bac926F primer	AAACTYAAAKGAATTGACG
	*

	1418 … 1432
16S rRNA *B. bifidum* CP010412	GTACACACCGCCCGT
16S rRNA *S. Epidermis* NR_074995	GTACACACCGCCCGT
16S rRNA *E. cloacae* CP012165	GTACACACCGCCCGT
16S rRNA *E. faecalis* CP014949	GTACACACCGCCCGT
16S rRNA *B. infantis* M58738.1	GTACACACNGCCCGT
16S rRNA *B. longum* ATCC 156697	GTACACACCGCCCGT
bac1394R primer	GYACACACCGCCCGT
