# Supplementary material for: Optimisation of 16S rRNA gut microbiota profiling of extremely low birth weight infants
Source: BMC Genomics. 2017 Nov 2;18:841. doi: 10.1186/s12864-017-4229-x (PMC5668952; doi:10.1186/s12864-017-4229-x)
Supplement: Supplementary file 15 — Melting curves of PCR amplicons from probiotic strains and bacterial preterm isolates. a Melting curves of PCR amplicons from probiotic strains (Bifidobacterium bifidum and Lactobacillus acidophilus) and bacterial preterm isolates (Enterococcus faecium and Streptococcus infantarius). Primers used to generate these amplicons were 530F-926R targeting region (V4 + V5). Bifidobacterium bifidum displayed the highest melting temperature. b Melting curve of PCR amplicon obtained from a mixed DNA sample (5 ng of Bifidobacterium bifidum, 5 ng of Lactobacillus acidophilus, 5 ng of Enterococcus faecium and 5 ng of Streptococcus infantarius). Primers used to generate these amplicons were 530F-926R targeting region (V4 + V5). Peak name (2) presents a melting temperature (Tm) similar to the melting temperature (Tm) obtained for B. bifidum. (PDF 100 kb) [file 12864_2017_4229_MOESM15_ESM.pdf]

a Amplicon melting curves from single bacterial strains

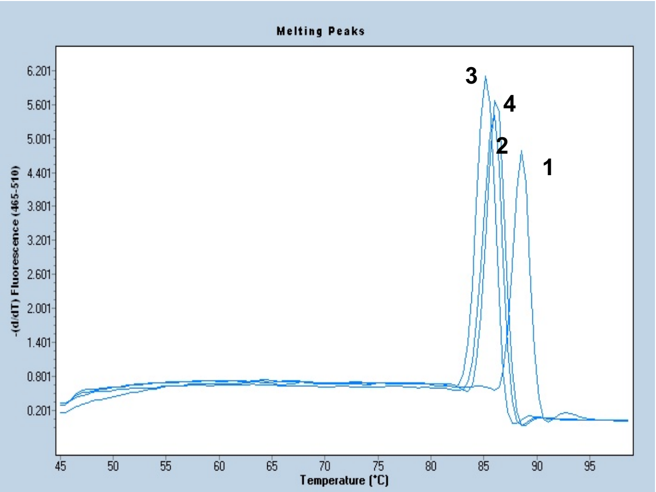

1	***Bifidobacterium bifidum*** **(Infloran isolate)**	**Tm (°C)= 88.60**
2	***Lactobacillus acidophilus*** **(Infloran isolate)**	**Tm (°C)= 85.90**
3	***Streptococcus infantarius*** **(preterm isolate)**	**Tm (°C)= 85.24**
4	***Enterococcus faecium*** **(preterm isolate)**	**Tm (°C)= 86.20**

b Amplicon melting curve from a mixed DNA sample

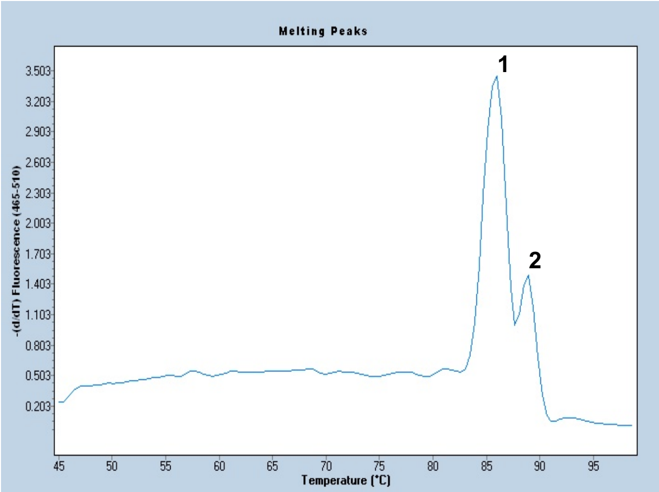

Peak 1 Tm (°C)= 85.89
Peak 2 Tm (°C)= 88.93
