# Supplementary material for: Optimisation of 16S rRNA gut microbiota profiling of extremely low birth weight infants
Source: BMC Genomics. 2017 Nov 2;18:841. doi: 10.1186/s12864-017-4229-x (PMC5668952; doi:10.1186/s12864-017-4229-x)
Supplement: Supplementary file 19 — Number of reads detected by PE and QIIME pipelines for the different hypervariable regions. (PDF 181 kb) [file 12864_2017_4229_MOESM19_ESM.pdf]

Additional file 19 - Number of reads detected by PE and QIIME pipelines for the different hypervariable regions

PE PROTOCOL

#Datasets	AP1E.27F	AP5D.27F	AP8C.27F	AP25E.27F	P29F.27F	P30N.27F	P31B.27F	P35C.27F	V2A.27F	V3J.27F	AP1E.530F	AP5D.530F	AP8C.530F	AP25E.530F	P29F.530F	P30N.530F	P31B.530F	P35C.530F	V2A.530F	V3J.530F	AP1E.926F	AP5D.926F	AP8C.926F	AP25E.926F	P29F.926F	P30N.926F	P31B.926F	P35C.926F	V2A.926F	V3J.926F
Bacteroides	74173	240	181	192	203	256	210	177	4084	225	87074	766	732	468	689	847	685	548	28415	588	64580	231	214	208	207	286	111	202	2188	243
Staphylococcus	17209	27009	11186	550	7318	1263	36532	23117	461	397	13872	32242	13985	1183	18988	4780	23928	17872	1171	879	25325	29048	11546	247	7289	2036	6709	12691	415	386
Lachnoclostridium	3645	23	19	32	20	28	29	18	22	20	3012	69	69	30	64	76	43	63	61	36	3572	30	27	0	22	16	0	0	19	17
Corynebacterium	696	724	13	0	14	13	87	0	0	17	12	0	0	0	0	0	0	0	12	0	2534	487	21	13	22	30	33	12	37	38
Enterococcus	517	71921	49529	465	34298	94964	832	789	649	686	1021	65588	45299	757	51896	90321	2042	4095	1827	1897	546	64246	39439	523	21281	88643	499	762	868	750
Bifidobacterium	353	561	496	394	37453	1941	15039	466	90378	85701	47	95	88	88	587	120	412	77	6939	6687	398	719	677	548	45983	3554	24655	576	87431	60377
Enterobacteriaceae	318	435	18567	105196	4550	1285	310	289	1232	491	242	782	12149	101384	8707	1491	382	516	3035	1075	372	566	21593	104877	7389	1407	309	456	2419	1429
Lactobacillus	182	399	302	295	2109	1572	392	70299	260	2005	430	2206	1756	970	9588	5408	1208	80373	429	6848	235	502	416	225	3003	2977	232	64811	377	1603
Streptococcus	139	410	7735	151	265	312	49657	225	8114	15475	583	1276	16110	383	1766	1625	77264	1230	42481	76928	131	303	10455	133	384	358	12090	240	6753	12543
Erysipelatoclostridiu	56	0	0	0	0	0	0	0	0	0	331	12	12	0	12	11	0	0	0	0	634	0	0	0	0	0	0	0	0	0
Acinetobacter	29	16	23	26	20	50	21	7921	19	23	0	12	17	49	21	23	0	1549	26	13	0	0	0	0	0	0	0	130	0	0
Prevotella	14	0	0	0	0	0	0	0	0	692	23	0	0	0	0	0	0	0	38	2442	0	0	0	0	0	0	0	0	11	867
Fusobacterium	0	0	0	0	0	0	0	0	0	0	0	0	0	0	0	0	0	0	11	0	0	0	0	0	0	0	0	0	0	0
Rhizobium	0	0	0	0	0	0	0	0	0	0	0	0	0	0	0	0	0	0	18	0	0	0	0	0	0	0	0	0	0	0
Curvibacter	0	0	0	0	0	0	0	0	0	0	0	0	0	0	0	0	30	0	0	0	0	0	0	0	0	0	0	0	0	0
Neisseria	0	0	17	0	0	0	0	0	0	0	0	0	31	0	0	0	0	0	0	0	0	0	35	0	0	0	0	0	0	0
Haemophilus	0	0	1393	0	0	0	0	0	0	401	13	29	2854	194	67	44	18	25	41	5438	13	22	2252	33	24	29	13	15	24	2733
Pseudomonas	0	0	0	0	0	0	0	364	0	0	0	0	0	0	0	0	0	108	0	0	0	0	0	0	0	0	0	142	0	0
Actinomyces	0	0	0	0	0	0	0	0	285	0	41	68	67	46	71	82	58	50	9315	99	0	0	0	0	0	0	0	0	1576	20
Varibaculum	0	0	0	0	0	0	0	0	0	0	0	0	0	0	0	0	0	0	16	0	0	0	0	0	0	0	0	0	0	0
Gardnerella	0	0	0	0	0	0	0	0	0	0	0	0	0	0	0	0	0	0	0	0	0	0	0	0	0	0	26	0	0	30
Rothia	0	0	0	0	0	0	0	0	0	0	0	0	0	0	0	0	0	0	0	0	0	0	0	0	0	0	0	0	93	138
Propionibacterium	0	15	56	0	0	0	24	0	0	0	0	20	110	34	0	0	83	16	27	70	0	27	231	0	0	0	17	0	0	0

**Bacillus**	0	0	0	0	0	0	33	0	0	0	0	0	0	0	0	0	0	0	0	0	0	0	0	0	0	0	0	0	0	0
**Gemella**	0	0	0	0	0	0	0	0	26	0	0	26	25	0	24	14	12	12	220	26	0	0	0	0	0	0	0	0	35	0
**Salinicoccus**	0	0	0	0	0	0	0	0	0	0	0	0	0	0	0	0	0	0	0	0	25	0	0	0	0	0	0	0	0	0
**Desemzia**	0	0	0	0	0	0	0	0	0	0	0	16	0	0	0	0	14	29	0	0	0	0	0	0	0	0	0	0	0	0
**Dolosigranulum**	0	0	0	0	0	0	0	0	12	11	0	0	0	0	0	0	0	0	0	0	0	0	0	0	0	0	0	0	18	40
**Granulicatella**	0	0	0	0	0	0	0	0	137	0	0	131	155	0	140	92	17	39	944	11	0	0	0	0	0	0	0	0	46	0
**Melissococcus**	0	0	0	0	0	0	0	0	0	0	0	13	0	0	0	0	0	0	0	0	0	0	0	0	0	0	0	0	0	0
**Mogibacterium**	0	0	0	0	0	0	0	0	0	0	0	0	0	0	0	0	0	0	70	0	0	0	0	0	0	0	0	0	17	0
**Lachnoanaerobacul**	0	0	0	0	0	0	0	0	17	0	19	0	0	0	0	0	0	0	399	0	0	0	0	0	0	0	0	0	27	0
**Stomatobaculum**	0	0	0	0	0	0	0	0	0	0	0	0	0	0	0	0	0	0	26	0	0	0	0	0	0	0	0	0	0	0
**Tyzzerella**	0	0	0	0	0	0	0	0	62	0	0	0	0	0	0	0	0	0	768	0	0	0	0	0	0	0	0	0	70	0
**Peptoclostridium**	0	0	0	0	0	0	0	0	0	0	0	0	25	0	0	0	0	0	0	0	0	0	0	0	0	0	0	0	0	0
**Peptostreptococcus**	0	0	0	0	0	0	0	0	0	0	0	0	0	0	0	0	0	0	67	0	0	0	0	0	0	0	0	0	0	0
**Scheffersomyces**	0	0	0	0	0	0	0	0	0	0	0	0	0	0	0	0	0	0	0	0	0	0	0	0	0	0	0	19	0	0
**Candida**	0	0	0	0	0	0	0	0	0	0	0	0	0	0	0	0	0	0	0	0	0	0	0	0	0	0	0	22	0	65
**Doassansia**	0	0	0	0	0	0	0	0	0	0	0	0	0	0	0	0	0	0	0	0	0	0	0	0	0	0	0	45	0	0
**Tilletiopsis**	0	0	0	0	0	0	0	0	0	0	0	0	0	0	0	0	0	0	0	0	0	0	0	0	0	0	60	53	0	0
**Conidiosporomyces**	0	0	0	0	0	0	0	0	0	0	0	0	0	0	0	0	0	0	0	0	0	0	0	0	0	0	29	0	0	0
**Tilletia**	0	0	0	0	0	0	0	0	0	0	0	0	0	0	0	0	0	0	0	0	0	0	0	0	0	0	21	0	0	0

QIIME PIPELINE

#Datasets	AP1E.27F	AP25E.27F	AP5D.27F	AP8C.27F	P30N.27F	P29F.27F	P31B.27F	P35C.27F	V2A.27F	V3J.27F	AP1E.530F	AP25E.530F	AP5D.530F	AP8C.530F	P30N.530F	P29F.530F	P31B.530F	P35C.530F	V2A.530F	V3J.530F	AP1E.926F	AP25E.926F	AP5D.926F	AP8C.926F	P30N.926F	P29F.926F	P31B.926F	P35C.926F	V2A.926F	V3J.926F
Bacteroides	18948	47	92	74	94	62	60	74	699	53	20830	108	177	175	196	159	161	129	6768	139	17067	42	56	56	68	52	56	63	690	92
Corynebacterium	480	2	366	5	8	4	36	0	0	3	216	6	77	5	6	6	44	3	47	40	627	3	77	5	7	3	14	4	6	11
Bifidobacterium	75	99	223	191	851	12380	5413	214	22830	22141	11	19	22	20	28	144	100	18	1693	1654	70	88	170	123	702	10317	13285	145	20156	16273
Enterobacteriaceae	53	25353	152	5956	1698	5445	80	122	330	116	58	24751	183	3119	631	5405	112	137	2336	639	94	23231	177	5696	696	6387	182	150	1304	917
Staphylococcus	19	0	0	0	0	0	0	0	0	0	3100	207	6624	3245	932	4191	5729	4124	249	192	6320	67	7489	2807	407	1933	3967	4233	108	118
Lactobacillus	12	32	44	32	223	251	40	13451	17	202	93	216	522	407	1329	2219	264	19120	120	1625	38	46	105	92	604	636	109	20298	89	543
Streptococcus	12	21	91	2149	65	57	12422	42	1362	2824	130	74	286	3989	366	405	18659	278	10197	18608	29	34	84	2468	109	108	7650	76	2348	5797
Paenibacillus	10	20	0	4	0	0	9	18	12	0	0	0	0	0	0	0	0	0	0	0	0	0	0	0	0	0	0	0	0	0
Acinetobacter	6	5	3	9	23	2	7	3442	3	3	1	11	2	4	4	4	1	369	5	3	0	9	1	584	2	3	2	308	5	3
Prevotella	1	0	0	0	0	0	1	0	0	154	5	1	1	1	2	1	1	2	9	589	0	1	0	0	1	0	2	0	2	327
Veillonella	1	0	236	1402	5	0	0	2	21	5	11	16	422	2235	46	36	24	32	893	215	4	5	222	1587	10	9	8	7	83	72
Finegoldia	1	1	7	1856	5	4	4	2	4	7	3	2	6	558	6	4	4	3	11	85	0	1	1	243	3	0	0	2	1	13
Porphyromonas	0	0	0	0	0	0	0	0	0	0	0	0	0	0	0	0	0	0	1	0	0	0	0	0	0	0	0	0	0	0
Fusobacterium	0	0	0	0	0	0	0	0	0	0	0	0	0	0	0	0	0	0	2	0	0	0	0	0	0	0	0	0	0	0
Agrobacterium	0	0	0	0	0	0	0	0	0	0	0	0	0	0	0	0	0	0	4	0	0	0	0	0	0	0	0	0	0	0
Enhydrobacter	0	0	0	0	0	0	0	0	0	0	0	0	0	0	0	0	0	0	4	0	0	0	0	0	0	0	0	0	3	0
Burkholderia	0	0	0	0	0	0	0	0	0	0	0	0	0	0	0	0	2	0	0	0	0	0	0	0	0	0	0	0	0	0
Neisseria	0	0	0	4	0	0	0	0	0	0	0	0	0	7	0	0	0	0	1	0	0	0	0	8	0	0	0	0	0	0
Erwinia	0	0	0	0	0	0	0	0	0	0	0	18	0	11	0	0	0	0	0	0	0	0	0	31	0	1	0	0	0	0
Pantoea	0	0	0	0	0	0	0	0	0	0	0	1	0	16	0	0	0	0	0	0	0	0	0	2	0	0	0	0	0	0
Proteus	0	0	0	0	0	0	0	0	0	0	0	0	0	5	0	0	0	0	0	0	0	0	0	0	0	0	0	0	0	0
Providencia	0	0	0	0	0	0	0	0	0	0	0	0	0	0	0	0	0	0	0	0	0	0	0	3	0	0	0	0	0	0
Serratia	0	0	0	0	0	0	0	0	0	0	0	0	0	0	6	1	0	0	0	0	0	7	0	11	1	0	0	0	0	0
Haemophilus	0	2	2	448	2	1	0	0	0	86	3	45	5	680	9	14	4	5	9	1306	4	11	5	576	10	10	7	7	7	1170
Pseudomonas	0	1	0	0	0	0	0	52	0	0	0	1	0	0	0	1	0	26	0	1	0	0	0	0	0	0	0	29	0	0

**Stenotrophomonas**	0	0	0	0	0	0	0	0	0	0	0	0	1	0	0	0	1	0	1	0	0	0	1	0	0	0	0	0	0	0
**Actinomyces**	0	0	0	0	0	0	0	0	52	0	10	10	15	15	19	16	13	11	2281	23	0	0	2	1	1	0	0	1	452	6
**Varibaculum**	0	0	0	0	0	0	0	0	0	0	0	0	0	0	0	0	0	0	4	0	0	0	0	0	0	0	0	0	0	0
**Microbacterium**	0	0	0	0	0	0	0	0	0	0	0	0	0	0	0	0	0	0	1	0	0	0	0	0	0	0	0	0	0	0
**Rothia**	0	0	0	0	0	0	0	0	0	1	0	0	0	0	0	0	0	0	0	0	0	0	1	0	0	0	0	0	26	45
**Propionibacterium**	0	0	3	20	0	0	9	2	0	0	1	8	4	26	0	1	20	4	6	17	0	0	5	46	0	0	8	2	0	2
**Atopobium**	0	0	0	0	0	0	0	0	4	0	0	0	1	0	0	1	1	0	39	1	0	0	0	0	0	0	0	0	11	0
**Anaerobacillus**	0	0	0	0	0	0	0	0	0	0	0	0	0	3	1	1	0	0	0	0	0	0	0	0	0	0	0	0	0	0
**Bacillus**	0	0	10	0	0	0	6	2	0	4	0	0	0	0	0	0	0	0	0	0	0	0	0	0	0	0	0	0	0	0
**Gemella**	0	0	0	0	0	0	0	0	0	0	0	0	0	0	0	0	0	0	17	2	0	0	0	0	0	0	0	0	3	2
**Listeria**	0	0	0	0	0	0	0	0	0	0	0	0	2	0	0	0	0	0	0	0	0	0	0	0	0	0	0	0	0	0
**Salinicoccus**	0	0	0	0	0	0	0	0	0	0	0	0	5	0	1	1	0	3	0	0	0	0	0	0	0	0	0	0	0	0
**Alloiococcus**	0	0	0	0	0	0	0	0	0	0	0	0	0	0	0	0	0	0	0	0	0	0	0	0	0	0	0	0	7	16
**Carnobacterium**	0	0	0	0	0	0	0	0	0	0	0	0	0	0	0	0	0	0	0	0	0	0	13	0	0	0	0	0	0	0
**Granulicatella**	0	0	0	0	0	0	0	0	22	0	44	0	348	53	1420	50	3	77	23	12	0	0	0	1	0	2	0	0	64	0
**Enterococcus**	0	0	125	0	35	21	0	0	0	0	178	141	15225	10796	19900	12361	466	1147	558	426	49	103	6351	9481	9350	2472	80	379	164	81
**Lactococcus**	0	0	0	0	0	0	0	0	0	0	0	0	0	0	0	0	0	0	0	0	116	16	10828	1779	13748	3712	300	1	158	189
**Clostridium**	0	0	0	0	0	0	0	0	14	0	1	1	2	3	1	2	2	2	186	2	0	0	0	0	0	0	0	1	23	0
**Mogibacterium**	0	0	0	0	0	0	0	0	1	0	0	0	0	0	0	0	0	0	17	0	0	0	0	0	0	0	0	0	6	0
**Butyrivibrio**	0	0	0	0	0	0	0	0	0	0	0	0	0	0	0	0	0	0	4	0	0	0	0	0	0	0	0	0	0	0
**Moryella**	0	0	0	0	0	0	0	0	0	0	0	0	0	0	0	0	0	0	2	0	0	0	0	0	0	0	0	0	0	0
**Peptostreptococcus**	0	0	0	0	0	0	0	0	0	0	0	0	1	1	0	0	1	0	17	0	0	0	0	0	0	0	0	0	0	0
**Epulopiscium**	0	0	0	0	0	0	0	0	0	0	0	0	0	0	0	0	0	0	1	0	0	0	0	0	0	0	0	0	0	0
**Dialister**	0	0	0	0	0	0	0	0	0	0	0	0	0	1	0	0	0	0	2	3	0	0	0	0	0	0	0	0	0	3
**Anaerococcus**	0	0	0	0	0	0	0	0	1	7	0	0	0	1	1	0	0	0	8	70	0	0	0	0	0	0	0	0	1	35
**Peptoniphilus**	0	0	0	0	0	0	0	0	0	1	0	0	0	1	0	0	1	0	11	47	0	0	0	0	0	0	0	0	0	12
