# Supplementary material for: Optimisation of 16S rRNA gut microbiota profiling of extremely low birth weight infants
Source: BMC Genomics. 2017 Nov 2;18:841. doi: 10.1186/s12864-017-4229-x (PMC5668952; doi:10.1186/s12864-017-4229-x)
Supplement: Supplementary file 20 — Number of reads shotgun and 16S rRNA data (PE versus QIIME). (PDF 286 kb) [file 12864_2017_4229_MOESM20_ESM.pdf]

Additional file 20 - Number of reads shotgun and 16S rRNA data (PE versus QIIME)

Comparison shotgun versus PE

#Datasets	AP8C.Shotgun	AP8C.27F	AP8C.530F	AP8C.926F
Enterococcus	64799	66818	61100	53203
Streptococcus	9656	10436	21730	14103
Staphylococcus	9426	15091	18863	15579
Veillonella	7200	0	0	0
Klebsiella	7093	0	0	0
Finegoldia	1652	0	0	0
Haemophilus	1035	1880	3850	3038
Salmonella	640	0	0	0
Propionibacterium	538	76	149	311
Shigella	500	0	0	0
Escherichia	317	18	57	0
Enterobacter	277	356	728	661
Cronobacter	191	0	0	0
Chlamydia	127	0	0	0
Cutibacterium	102	0	0	0
Bacillus	101	0	0	0
Listeria	93	0	0	0
Lactobacillus	91	407	2367	561
Pantoea	81	0	0	0
Citrobacter	79	0	264	92
Yersinia	67	0	0	0

#Datasets	P29F. Shotgun	P29.27F	P29F.530F	P29F.926F
Bifidobacterium	69180	39679	619	48726
Enterococcus	12998	36337	54696	22550
Enterobacter	7517	19444	13766	15417
Staphylococcus	1642	7751	20012	7724
Lactobacillus	1388	2235	10105	3183
Klebsiella	432	0	0	0
Mycobacterium	373	0	0	0
Shigella	318	0	0	0
Salmonella	315	0	0	0
Streptococcus	308	280	1859	407
Escherichia	266	265	62	0
Chlamydia	148	0	0	0
Cronobacter	130	0	0	0
Vibrio	71	0	0	0
Microbacterium	63	0	0	0
Streptomyces	49	0	0	0
Bordetella	45	0	0	0
Citrobacter	44	19	264	19
Gardnerella	37	0	0	0
Serratia	29	0	0	0
Yersinia	29	0	0	0

#Datasets	V3J.Shotgun	V3J.27F	V3J.530F	V3J.926F
Bifidobacterium	138083	128700	10029	90556
Streptococcus	4283	23239	115371	18815
Mycobacterium	821	0	0	0
Lactobacillus	521	3010	10271	2404
Prevotella	196	1040	3663	1300
Rothia	160	0	0	207
Escherichia	152	66	97	137
Haemophilus	125	602	8156	4100
Streptomyces	114	0	0	0
Chlamydia	111	0	0	0
Gardnerella	100	0	0	46
Actinomyces	85	0	149	30
Corynebacterium	77	26	0	56
Veillonella	66	0	0	0
Shigella	57	0	19	169
Eggerthella	51	0	0	0
Bordetella	39	0	0	0
Enterococcus	29	1030	2845	1126
Vibrio	28	0	0	0
Georgenia	27	0	0	0
Nocardia	25	0	0	0

**Serratia**	62	0	0	0
**Vibrio**	59	0	0	0
**Raoultella**	48	0	0	0
**Carnobacterium**	47	0	0	0
**Neisseria**	44	23	42	47
**Sep1virus**	40	0	0	0
**Pseudomonas**	26	0	0	0
**Homo**	26	0	0	0
**Bifidobacterium**	25	670	119	913
**Mycobacterium**	25	0	0	0
**Acinetobacter**	21	31	23	0
**Paenibacillus**	21	0	0	0
**Phifelvirus**	18	0	0	0
**Clostridium**	17	0	0	0
**Peptoniphilus**	17	0	0	0
**Xenorhabdus**	16	0	0	0
**Thelohanellus**	13	0	0	0
**Sextaecvirus**	13	0	0	0
**Fusobacterium**	12	0	0	0
**Bordetella**	12	0	0	0
**Providencia**	11	0	0	0
**Ruminiclostridium**	11	0	0	0
**Megasphaera**	10	0	0	0
**Kosakonia**	9	0	0	0
**Aggregatibacter**	9	0	0	0
**Heliobacterium**	9	0	0	0
**Anaerococcus**	9	0	0	0
**Acholeplasma**	9	0	0	0

**Listeria**	28	0	0	0
**Eggerthella**	26	0	0	0
**Rothia**	22	0	0	0
**Pseudomonas**	18	0	0	0
**Corynebacterium**	17	15	0	23
**Bacillus**	17	0	0	0
**Pantoea**	16	0	0	0
**Actinomyces**	15	0	75	0
**Clostridium**	14	0	0	0
**Burkholderia**	13	0	0	0
**Bacteroides**	0	216	727	220
**Haemophilus**	0	0	71	25
**Acinetobacter**	0	21	22	0
**Gemella**	0	0	25	0
**Granulicatella**	0	0	147	0
**Lachnoclostridium**	0	21	67	23
**Erysipelatoclostridium**	0	0	12	0

**Cutibacterium**	25	0	0	0
**Candida <Debaryomycetaceae>**	24	0	0	0
**Collinsella**	23	0	0	0
**Propionibacterium**	21	0	105	0
**Clostridium**	21	0	0	0
**Rhodococcus**	19	0	0	0
**Arthrobacter**	19	0	0	0
**Staphylococcus**	17	595	1319	579
**Anaerococcus**	17	0	0	0
**Bacteroides**	0	337	882	364
**Citrobacter**	0	69	2118	0
**Enterobacter**	0	96	208	150
**Salmonella**	0	0	0	27
**Acinetobacter**	0	34	20	0
**Gemella**	0	0	39	0
**Dolosigranulum**	0	17	0	59
**Granulicatella**	0	0	17	0
**Lachnoclostridium**	0	30	55	25
**Candida**	0	0	0	97

**Corynebacterium**	8	17	0	28
**Clostridioides**	8	0	0	0
**Erwinia**	7	0	0	0
**Streptomyces**	7	0	0	0
**Anoxybacillus**	7	0	0	0
**Eubacterium**	7	0	0	0
**Lagierella**	7	0	0	0
**Burkholderia**	6	0	0	0
**Edwardsiella**	6	0	0	0
**Brenneria**	6	0	0	0
**Yokenella**	5	0	0	0
**Trichuris**	5	0	0	0
**Wolbachia**	4	0	0	0
**Aeromonas**	4	0	0	0
**Photorhabdus**	4	0	0	0
**Proteus**	4	0	0	0
**Dickeya**	4	0	0	0
**Rhodanobacter**	4	0	0	0
**Microbacterium**	4	0	0	0
**Tyzzerella**	4	0	0	0
**Coprobacillus**	4	0	0	0
**Bacteroides**	3	244	988	290
**Ralstonia**	3	0	0	0
**Cedecea**	3	0	0	0
**Kluyvera**	3	0	0	0
**Legionella**	3	0	0	0
**Gallibacterium**	3	0	0	0
**Histophilus**	3	0	0	0

**Psychrobacter**	3	0	0	0
**Pseudoxanthomonas**	3	0	0	0
**Xanthomonas**	3	0	0	0
**Blautia**	3	0	0	0
**Peptostreptococcus**	3	0	0	0
**Nosema**	3	0	0	0
**Prevotella**	2	0	0	0
**Rhizobium**	2	0	0	0
**Azospirillum**	2	0	0	0
**Achromobacter**	2	0	0	0
**Candidatus Accumulibacter**	2	0	0	0
**Helicobacter**	2	0	0	0
**Pluralibacter**	2	0	0	0
**Trabulsiella**	2	0	0	0
**Obesumbacterium**	2	0	0	0
**Sodalis**	2	0	0	0
**Mannheimia**	2	0	0	0
**Photobacterium**	2	0	0	0
**Rothia**	2	0	0	0
**Gemella**	2	0	33	0
**Brevibacillus**	2	0	0	0
**Sporosarcina**	2	0	0	0
**Melissococcus**	2	0	0	0
**Leuconostoc**	2	0	0	0
**Lactococcus**	2	0	0	0
**Thermobrachium**	2	0	0	0
**Ruminococcus**	2	0	0	0
**Negativicoccus**	2	0	0	0

**Parvimonas**	2	0	0	0
**Ureaplasma**	2	0	0	0
**Beauveria**	2	0	0	0
**Macaca**	2	0	0	0
**Trichinella**	2	0	0	0
**Plasmopara**	2	0	0	0
**Arachis**	2	0	0	0
**Hydrogenophaga**	1	0	0	0
**Morococcus**	1	0	0	0
**Thauera**	1	0	0	0
**Campylobacter**	1	0	0	0
**Actinobacillus**	1	0	0	0
**Pasteurella**	1	0	0	0
**Xylella**	1	0	0	0
**Acidipropionibacterium**	1	0	0	0
**Collinsella**	1	0	0	0
**Massilibacterium**	1	0	0	0
**Planococcus**	1	0	0	0
**Aerococcus**	1	0	0	0
**Oribacterium**	1	0	0	0
**Desulfosporosinus**	1	0	0	0
**Peptoclostridium**	1	0	33	0
**Acidaminococcus**	1	0	0	0
**Selenomonas**	1	0	0	0
**Bos**	1	0	0	0
**Brachypodium**	1	0	0	0
**Kayvirus**	1	0	0	0
**Twortvirus**	1	0	0	0

**Actinomyces**	0	0	91	0
**Granulicatella**	0	0	211	0
**Lachnoclostridium**	0	25	93	36
**Erysipelatoclostridium**	0	0	17	0

Comparison shotgun versus QIIME

#Datasets	AP8C.27F	AP8C.530F	AP8C.926F	AP8C.shotgun
**Enterococcus**	0	10819	9502	11460
**Streptococcus**	2149	3998	2474	1781
**Staphylococcus**	0	3252	2814	1704
**Klebsiella**	0	2	34	1282
**Veillonella**	1402	2240	1590	1276
**Finegoldia**	1856	559	243	291
**Haemophilus**	448	682	578	190
**Salmonella**	0	0	0	138
**Propionibacterium**	20	26	47	98
**Shigella**	0	0	0	92
**Escherichia**	0	0	0	90
**Enterobacter**	0	0	0	55
**Cronobacter**	0	0	0	39
**Lactobacillus**	32	408	93	34
**Bacillus**	0	0	0	29
**Cutibacterium**	0	0	0	25
**Chlamydia**	0	0	0	24
**Citrobacter**	0	0	35	22

#Datasets	P29F.27F	P29F.530F	P29F.926F	P29F.shotgun
**Bifidobacterium**	16904	197	14088	21153
**Lactobacillus**	343	3030	869	424
**Bacteroides**	85	217	71	0
**Streptococcus**	78	553	148	94
**Enterococcus**	28	16878	3375	3975
**Enterobacter**	7	0	67	2299
**Finegoldia**	6	5	0	0
**Corynebacterium**	5	9	4	5
**Acinetobacter**	3	5	5	0
**Haemophilus**	2	19	14	0
**Prevotella**	0	2	0	0
**Bordetella**	0	0	0	13
**Burkholderia**	0	0	0	4
**Citrobacter**	0	0	4	13
**Cronobacter**	0	0	0	42
**Escherichia**	0	0	0	82
**Klebsiella**	0	1	0	133
**Salmonella**	0	0	0	98

#Datasets	V3J.shotgun	V3J.27F	V3J.530F	V3J.926F
**Bifidobacterium**	39256	39844	2976	29285
**Streptococcus**	1219	5082	33485	10433
**Lactobacillus**	148	364	2924	976
**Prevotella**	56	277	1061	589
**Haemophilus**	35	155	2350	2106
**Bacteroides**	0	95	251	166
**Finegoldia**	0	13	153	24
**Anaerococcus**	5	12	126	63
**Veillonella**	19	9	386	130
**Bacillus**	0	8	0	0
**Acinetobacter**	0	6	5	5
**Corynebacterium**	22	5	72	20
**Rothia**	45	2	0	81
**Peptoniphilus**	0	2	85	22
**Bordetella**	11	0	0	0
**Escherichia**	43	0	0	0
**Shigella**	16	0	0	0
**Trabulsiella**	0	0	0	3

**Listeria**	0	0	0	20
**Serratia**	0	1	11	19
**Yersinia**	0	0	0	19
**Pantoea**	0	16	2	17
**Neisseria**	4	7	8	16
**Vibrio**	0	0	0	14
**Carnobacterium**	0	0	0	13
**Raoultella**	0	0	0	11
**Pseudomonas**	0	1	0	10
**Acinetobacter**	9	4	585	9
**Clostridium**	0	3	0	8
**Xenorhabdus**	0	0	0	7
**Homo**	0	0	0	7
**Sep1virus**	0	0	0	7
**Burkholderia**	0	0	0	6
**Bifidobacterium**	191	20	124	6
**Mycobacterium**	0	0	0	6
**Ruminiclostridium**	0	0	0	6
**Peptoniphilus**	0	1	0	6
**Fusobacterium**	0	1	0	5
**Edwardsiella**	0	0	0	5
**Providencia**	0	0	3	5
**Paenibacillus**	4	0	0	5
**Phifelvirus**	0	0	0	5
**Kosakonia**	0	0	0	4
**Erwinia**	0	11	31	4
**Dickeya**	0	0	0	4
**Aggregatibacter**	0	0	0	4

**Shigella**	0	0	0	97
**Erwinia**	0	0	2	0
**Pantoea**	0	0	0	5
**Proteus**	0	1	0	0
**Serratia**	0	1	0	9
**Yersinia**	0	0	0	9
**Pseudomonas**	0	1	0	6
**Vibrio**	0	0	0	22
**Chlamydia**	0	0	0	45
**Actinomyces**	0	21	0	5
**Gardnerella**	0	0	0	11
**Mycobacterium**	0	0	0	115
**Microbacterium**	0	0	0	19
**Rothia**	0	0	0	6
**Propionibacterium**	0	1	0	0
**Streptomyces**	0	0	0	15
**Atopobium**	0	1	0	0
**Eggerthella**	0	0	0	7
**Anaerobacillus**	0	1	0	0
**Bacillus**	0	1	0	5
**Listeria**	0	0	0	8
**Salinicoccus**	0	1	0	0
**Staphylococcus**	0	5723	2640	503
**Granulicatella**	0	68	3	0
**Lactococcus**	0	0	5069	0
**Clostridium**	0	2	0	4
**Veillonella**	0	49	12	0

**Erwinia**	0	0	1	0
**Pseudomonas**	0	0	1	0
**Vibrio**	8	0	0	0
**Chlamydia**	32	0	0	0
**Actinomyces**	24	0	42	11
**Gardnerella**	28	0	0	0
**Mycobacterium**	234	0	0	0
**Nocardia**	7	0	0	0
**Rhodococcus**	5	0	0	0
**Georgenia**	8	0	0	0
**Arthrobacter**	5	0	0	0
**Cutibacterium**	7	0	0	0
**Propionibacterium**	6	0	30	4
**Streptomyces**	32	0	0	0
**Atopobium**	0	0	1	0
**Collinsella**	7	0	0	0
**Eggerthella**	14	0	0	0
**Gemella**	0	0	4	3
**Staphylococcus**	5	0	346	212
**Alloiococcus**	0	0	0	28
**Granulicatella**	0	0	22	0
**Enterococcus**	8	0	767	145
**Lactococcus**	0	0	0	340
**Clostridium**	6	0	4	0
**Peptostreptococcus**	0	0	1	0
**Dialister**	0	0	6	5
**Candida <Debaryomycetaceae>**	7	0	0	0

**Anaerococcus**	0	1	0	4
**Bordetella**	0	0	0	3
**Ralstonia**	0	0	0	3
**Aeromonas**	0	0	0	3
**Cedecea**	0	0	0	3
**Kluyvera**	0	0	0	3
**Proteus**	0	5	0	3
**Xanthomonas**	0	0	0	3
**Corynebacterium**	5	5	5	3
**Clostridioides**	0	0	0	3
**Megasphaera**	0	0	0	3
**Acholeplasma**	0	0	0	3
**Prevotella**	0	1	0	2
**Rhizobium**	0	0	0	2
**Azospirillum**	0	0	0	2
**Candidatus Accumulibacter**	0	0	0	2
**Helicobacter**	0	0	0	2
**Trabulsiella**	0	0	14	2
**Yokenella**	0	0	0	2
**Photorhabdus**	0	0	0	2
**Brenneria**	0	0	0	2
**Sodalis**	0	0	0	2
**Legionella**	0	0	0	2
**Gallibacterium**	0	0	0	2
**Mannheimia**	0	0	0	2
**Psychrobacter**	0	0	0	2
**Photobacterium**	0	0	0	2
**Rhodanobacter**	0	0	0	2

**Pseudoxanthomonas**	0	0	0	2
**Microbacterium**	0	0	0	2
**Rothia**	0	0	0	2
**Streptomyces**	0	0	0	2
**Anoxybacillus**	0	0	0	2
**Gemella**	0	0	0	2
**Sporosarcina**	0	0	0	2
**Leuconostoc**	0	0	0	2
**Eubacterium**	0	0	0	2
**Heliobacterium**	0	0	0	2
**Blautia**	0	0	0	2
**Tyzzerella**	0	0	0	2
**Peptostreptococcus**	0	1	0	2
**Ruminococcus**	0	0	0	2
**Coprobacillus**	0	0	0	2
**Parvimonas**	0	0	0	2
**Ureaplasma**	0	0	0	2
**Macaca**	0	0	0	2
**Trichinella**	0	0	0	2
**Thelohanellus**	0	0	0	2
**Arachis**	0	0	0	2
**Sextaecvirus**	0	0	0	2
**Bacteroides**	74	175	56	1
**Wolbachia**	0	0	0	1
**Achromobacter**	0	0	0	1
**Hydrogenophaga**	0	0	0	1
**Morococcus**	0	0	0	1
**Thauera**	0	0	0	1

**Campylobacter**	0	0	0	1
**Pluralibacter**	0	0	0	1
**Obesumbacterium**	0	0	0	1
**Actinobacillus**	0	0	0	1
**Histophilus**	0	0	0	1
**Pasteurella**	0	0	0	1
**Xylella**	0	0	0	1
**Acidipropionibacterium**	0	0	0	1
**Collinsella**	0	0	0	1
**Massilibacterium**	0	0	0	1
**Brevibacillus**	0	0	0	1
**Planococcus**	0	0	0	1
**Aerococcus**	0	0	0	1
**Melissococcus**	0	0	0	1
**Lactococcus**	0	0	1783	1
**Thermobrachium**	0	0	0	1
**Oribacterium**	0	0	0	1
**Desulfosporosinus**	0	0	0	1
**Peptoclostridium**	0	0	0	1
**Acidaminococcus**	0	0	0	1
**Selenomonas**	0	0	0	1
**Negativicoccus**	0	0	0	1
**Lagierella**	0	0	0	1
**Beauveria**	0	0	0	1
**Nosema**	0	0	0	1
**Bos**	0	0	0	1
**Trichuris**	0	0	0	1
**Plasmopara**	0	0	0	1

**Brachypodium**	0	0	0	1
**Kayvirus**	0	0	0	1
**Twortvirus**	0	0	0	1
**Enhydrobacter**	0	1	0	0
**Actinomyces**	0	15	1	0
**Atopobium**	0	1	0	0
**Anaerobacillus**	0	3	0	0
**Salinicoccus**	0	1	0	0
**Granulicatella**	0	53	1	0
**Mogibacterium**	0	1	0	0
**Moryella**	0	1	0	0
**Epulopiscium**	0	1	0	0
**Dialister**	0	1	0	0
